# Lamin A/C phosphorylation at serine 22 is a conserved heat shock response to regulate nuclear adaptation during stress

**DOI:** 10.1242/jcs.259788

**Published:** 2023-02-27

**Authors:** Laura Virtanen, Emilia Holm, Mona Halme, Gun West, Fanny Lindholm, Josef Gullmets, Juho Irjala, Tiina Heliö, Artur Padzik, Annika Meinander, John E. Eriksson, Pekka Taimen

**Affiliations:** ^1^ Institute of Biomedicine and FICAN West Cancer Centre, University of Turku, 20520 Turku, Finland; ^2^ Faculty of Science and Engineering, Åbo Akademi University, 20520 Turku, Finland; ^3^ Heart and Lung Center, Helsinki University Hospital and University of Helsinki, 00029 Helsinki, Finland; ^4^ Genome Editing Core, Turku Bioscience Centre, University of Turku and Åbo Akademi University, 20520 Turku, Finland; ^5^ Turku Bioscience Centre, University of Turku and Åbo Akademi University, 20520 Turku, Finland; ^6^ Department of Pathology, Turku University Hospital, 20520 Turku, Finland

**Keywords:** Heat shock, Heat shock response, Lamin A/C, Phosphorylation, Lap2α

## Abstract

The heat shock (HS) response is crucial for cell survival in harmful environments. Nuclear lamin A/C, encoded by the *LMNA* gene, contributes towards altered gene expression during HS, but the underlying mechanisms are poorly understood. Here, we show that upon HS, lamin A/C was reversibly phosphorylated at serine 22 in concert with HSF1 activation in human cells, mouse cells and *Drosophila melanogaster in vivo*. Consequently, the phosphorylation facilitated nucleoplasmic localization of lamin A/C and nuclear sphericity in response to HS. Interestingly, lamin A/C knock-out cells showed deformed nuclei after HS and were rescued by ectopic expression of wild-type lamin A, but not by a phosphomimetic (S22D) lamin A mutant. Furthermore, HS triggered concurrent downregulation of lamina-associated protein 2α (Lap2α, encoded by *TMPO*) in wild-type lamin A/C-expressing cells, but a similar response was perturbed in lamin A/C knock-out cells and in *LMNA* mutant patient fibroblasts, which showed impaired cell cycle arrest under HS and compromised survival at recovery. Taken together, our results suggest that the altered phosphorylation stoichiometry of lamin A/C provides an evolutionarily conserved mechanism to regulate lamina structure and serve nuclear adaptation and cell survival during HS.

## Teaser

**Highlighted Article:** Phosphorylation of nuclear lamin A/C at Ser22 is an evolutionarily conserved mechanism that regulates lamin dynamics to serve nuclear adaptation and cell survival in heat-shocked cells.

## INTRODUCTION

The heat shock (HS) response is an evolutionarily conserved mechanism and vital for cell survival in harmful environments or pathophysiological stresses such as high temperature, fever or inflammation (Åkerfelt et al., 2010). Exposure to stressful conditions leads to synthesis of heat shock proteins (HSPs) that have a key role in protecting cellular homeostasis (Lindquist, 1986). The expression of HSP genes during HS is mainly controlled by heat shock factor 1 (HSF1) (Wu, 1995). Upon stress, inactive HSF1 monomers are converted to an active trimer that is hyperphosphorylated and translocated into the nucleus. Active HSF1 binds to heat shock elements at the promoter regions of HSP genes and induces transcription (Åkerfelt et al., 2010). The transcriptional response to HS results in the activation of hundreds and repression of thousands of genes (Mahat et al., 2016). HSF1 is critical for the induction of HSPs and over 200 other genes; however, the induction and repression of transcription during HS are comprehensive and the majority of these changes are HSF1 independent (Mahat et al., 2016). The DNA-binding activity of HSF1 can be detected within minutes of HS and is maintained at high levels for an hour. Thereafter, the DNA-binding activity of HSF1 decreases to control levels, even if the cells are still exposed to the HS (Kline and Morimoto, 1997).

Accumulating data show that the nuclear lamina is dynamically remodeled in response to environmental cues. The ratio of A-type lamins (primarily lamins A and C, encoded by *LMNA*) and B-type lamins (lamins B1 and B2) correlates with tissue stiffness, and the elasticity and organization of the lamina is further regulated by the phosphorylation or dephosphorylation status of lamins (Kochin et al., 2014; Buxboim et al., 2014; Swift et al., 2013). There are a number of studies suggesting that altered dynamics of lamins contribute to and are beneficial for cellular adaptation under HS. Early studies reported heat-induced stabilization of the nucleoskeleton and dephosphorylation of lamin A/C during HS (Krachmarov and Traub, 1993). Several studies have also demonstrated that lamin B1 is upregulated upon HS (Dynlacht et al., 1999; Pradhan et al., 2020; Zhu et al., 1999) and downregulated during recovery (Haddad and Paulin-Levasseur, 2008). In *Drosophila* Schneider 2 cells, lamin Dm2 is dephosphorylated to lamin Dm1 during HS (Smith et al., 1987). Furthermore, hypersensitivity to HS has been reported in dermal fibroblasts obtained from Hutchinson–Gilford progeria syndrome (HGPS) and familial partial lipodystrophy (FPLD) patients carrying G608G and R482Q/W mutations in the lamin A/C gene (*LMNA*), respectively (Paradisi et al., 2005; Vigouroux et al., 2001). Interestingly, a recent study demonstrated that HS upregulates lamin A/C and that lamin A/C is required for heat shock-mediated transcriptional induction of the Hsp70 genes (Pradhan et al., 2020).

As lamin A/C has been shown to contribute to the conserved HS response, we wanted to explore whether this response could be coupled to lamin A/C phosphorylation, which we have shown to be a primary determinant of lamin organization and assembly in interphase cells (Kochin et al., 2014). We show that lamin A/C was phosphorylated at serine 22 upon prolonged HS in both transformed and primary human cell lines, as well as in mouse fibroblasts and *Drosophila melanogaster in vivo*. Lamin A/C phosphorylation led to increased nucleoplasmic localization of lamin A/C and increased sphericity of the nucleus in human fibroblasts. Furthermore, data from lamin A/C knock-out (KO) and mutant cells demonstrated that a balanced lamin structure was needed to avoid nuclear deformation under HS. In addition, our results reveal that lamin A/C was required during prolonged HS to regulate the functions of the nuclear lamin A/C-binding partner, lamina-associated polypeptide 2 alpha (Lap2α, encoded by *TMPO*), which we show to determine cell cycle progression and cell survival during HS.

## RESULTS

### Lamin A/C is phosphorylated at serine 22 during HS

**Fig. 1.**
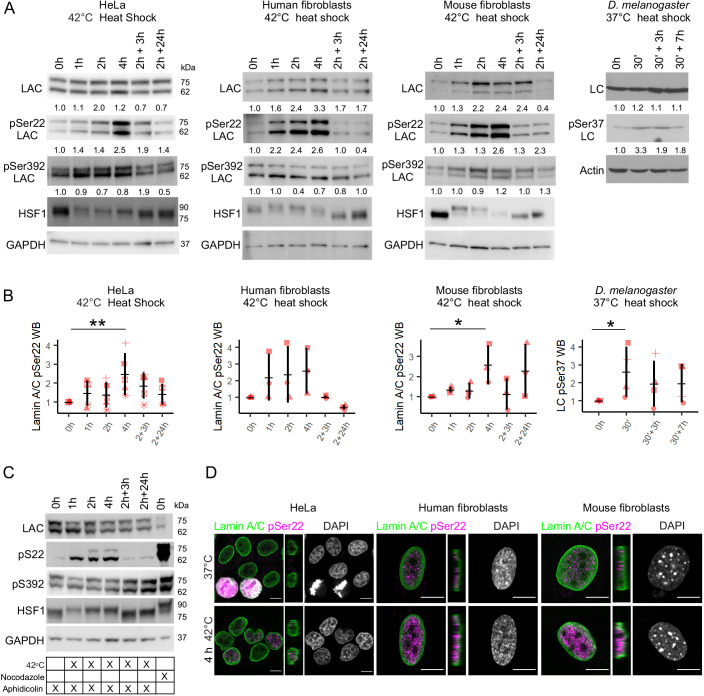
**Lamin A/C is phosphorylated at serine 22 upon HS.** (A) Western blot (WB) analysis of lamin A/C (LAC), phospho-serine 22 lamin A/C (pSer22 LAC), phospho-serine 392 lamin A/C (pSer392 LAC) and heat shock factor 1 (HSF1) after 1–4 h heat shock (HS) at 42°C, and 3 h and 24 h recovery at 37°C in HeLa cells and primary human and mouse fibroblasts. Whole *D. melanogaster* flies were heat-shocked for 30 min at 37°C and left to recover for 3 or 7 h at 22°C. The average numerical values of signal intensities relative to loading control (GAPDH or actin) from individual experiments are shown below each blot. pSer22 and pSer392 lamin A/C levels were normalized to GAPDH and lamin A/C. (B) Quantification of pSer22 lamin A/C WB intensities upon HS and recovery in HeLa cells (*n*=5), human fibroblasts, mouse fibroblasts and *D. melanogaster* fruit flies (*n*=4). Plots show mean±s.d. and the individual datapoints. The shapes of the datapoints indicate each individual replicate. *P<0.05; **P<0.01 (Mann–Whitney test). (C) Heat shock experiment with synchronized HeLa cells. Aphidicolin was used to synchronize cells to G1/S phase and nocodazole to synchronize cells to mitosis (positive control). GAPDH was used as a loading control (*n*=2). (D) HeLa cells, human and mouse fibroblasts were cultured either in normal culture conditions or exposed to 4 h HS at 42°C, fixed and stained for lamin A/C (green), pSer22 lamin A/C (magenta) and DAPI (gray). The narrow panels show the orthogonal views of the *yz*-plane. Scale bars: 10 μm.

To gain a better understanding of the function of the nuclear lamina under heat-induced stress, we examined the expression levels, localization and post-translational modifications of nuclear lamin A/C during HS. To this end, HeLa cells and primary human and mouse fibroblasts were exposed to HS for different periods of time (Fig. 1A,B). We used HSF1 gel shift as an established marker to indicate the HS response (Åkerfelt et al., 2010). Intriguingly, in concert with induced HS response, we observed gradually increased phosphorylation of lamin A/C at serine 22 (Ser22) upon 1–4 h HS at 42°C in all the cell lines (normalized to lamin A/C and GAPDH levels), whereas the phosphorylation levels of serine 392 remained relatively stable throughout the experiments (Fig. 1A). Whole *D. melanogaster* fruit flies that were heat-shocked for 30 min at 37°C showed similarly increased phosphorylation at Ser37, which is homologous to Ser22 in mammals (Fig. 1A,B). During the recovery phase, phosphorylated Ser22 (pSer22) levels were reduced along with the attenuation of the HS response, as indicated with the lost gel shift of HSF1 (Fig. 1A,B).

Additionally, we performed liquid chromatography coupled with mass spectrometry (LC-MS/MS) analyses to identify any additional differently phosphorylated residues in lamin A/C. Apart from Ser22, there were five phosphorylated residues (Thr3, Ser5, Ser268, Ser398 and Thr590) that were detected in the HS samples but not in the control samples (Table S1). To check how the HS-mediated Ser22 phosphorylation relates to the previously identified mitotically induced Ser22 phosphorylation, HeLa cells were arrested at the G1/S phase with aphidicolin prior to HS (Fig. 1C). Lamin A/C was found to be equally phosphorylated in synchronized cells upon HS, confirming that phosphorylation of lamin A/C at Ser22 takes place in heat-shocked interphase cells rather than in mitotic cells (Fig. 1C). In comparison, nocodazole-arrested mitotic control cells had significantly more pSer22 lamin A/C compared to the heat-shocked cells at the G1/S phase (Fig. 1C). Confocal microscopy analysis with an anti-pSer22 lamin A/C antibody also showed increased nucleoplasmic labeling in non-mitotic human and mouse cells upon HS (Fig. 1D). This analysis also showed that the phosphorylation level was significantly higher in mitotic cells in which all the lamins are in a disassembled state. Although the level of HS-induced phosphorylation was lower, the shift from the assembled form at the nuclear lamina to nucleoplasmic lamin was also more modest. In summary, these results indicate that lamin A/C phosphorylation at Ser22 is an evolutionarily conserved HS response mechanism, the onset and cessation of which occur in concert with HSF1 activation and attenuation.

### Phosphorylation of lamin A/C facilitates nuclear sphericity in response to severe HS

To study further how HS and lamin phosphorylation affect nuclear and lamina structure, HeLa cells, primary human dermal fibroblasts from a healthy individual and from a dilated cardiomyopathy patient carrying the p.S143P mutation in *LMNA*, as well as primary mouse fibroblasts were imaged and analyzed in detail upon HS. The morphological changes under 42°C HS were highly cell-type dependent. There were no detectable differences in lamin A/C staining intensities at the lamina or in the nucleoplasm between control and heat-shocked HeLa cells (Fig. S1A,B). However, the nuclear area and volume increased during HS, whereas the nuclear sphericity decreased (Fig. S1C–E). Mouse fibroblasts showed neither changes in nucleoplasm/lamina intensity ratios nor morphological alterations (Fig. S1F–J). In contrast, normal human fibroblasts showed a statistically significant decrease in lamin A/C staining intensity at the lamina under 42°C HS but exhibited no morphological changes during HS (Fig. S2).

**Fig. 2.**
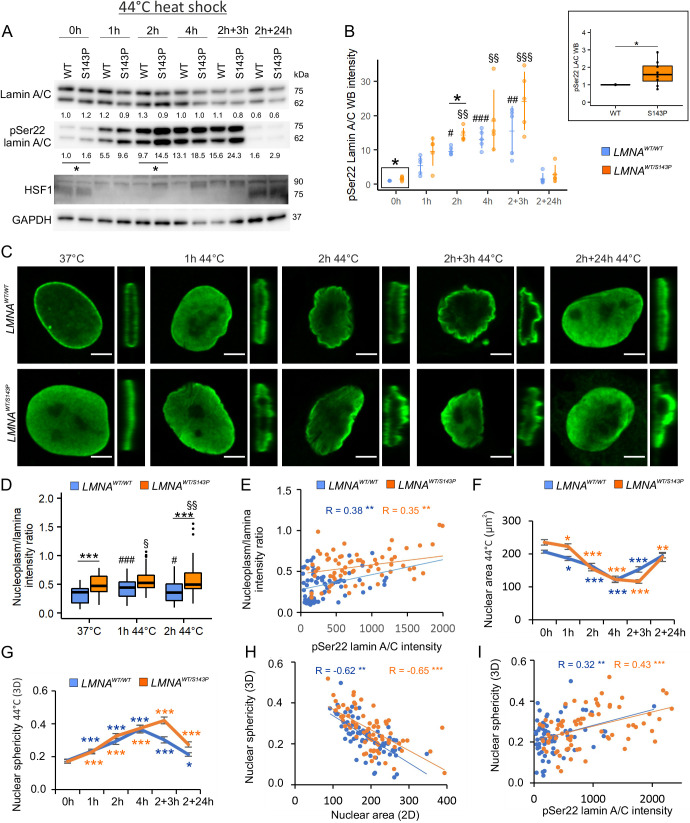
**Phosphorylation of lamin A/C correlates with nuclear sphericity in response to HS.** (A) Western blot analysis of control and patient fibroblasts carrying the p.S143P mutation in *LMNA* as detected with antbodies against lamin A/C, pSer22 lamin A/C and HSF1 upon 1–4 h HS at 44°C and at the recovery. The average numerical values of signal intensities relative to the loading control (GAPDH) are shown below each blot (*n*=5). pSer22 lamin A/C was normalized to GAPDH and lamin A/C. (B) Quantified pSer22 lamin A/C WB intensity in control and patient fibroblasts (*n*=8). The 0 h time point has been enlarged on the top right corner. *P<0.05 (Kruskal–Wallis/Dunn’s test). (C) Confocal microscopy images of *LMNA^WT/WT^* and *LMNA^WT/S143P^* fibroblasts stained for lamin A/C in normal culture conditions, after 1 h and 2 h HS at 44°C and after 3 h and 24 h recovery. The narrow panels show the orthogonal views of the *yz*-plane. Scale bars: 5 µm. (D) Lamin A/C fluorescence intensities at the lamina region and in the nucleoplasm were determined from the mid-plane confocal sections of randomly selected cells and the average ratios of the signals (nucleoplasm/lamina) were plotted (*n*=50, from three biological replicates). ***P<0.001; #, *LMNA^WT/WT^* control versus *LMNA^WT/WT^* HS; §, *LMNA^WT/S143P^* control versus *LMNA^WT/S143P^* HS (two-way ANOVA with Tukey's post hoc test). (E) Scatter plot of lamin A/C nucleoplasm/lamina intensity ratio versus pSer22 lamin A/C intensity determined from randomly selected cells at 37°C and after 1 h and 2 h HS at 44°C (*n*=70, two biological replicates). **P<0.01 (two-tailed unpaired *t*-test). (F,G) Nuclear area and nuclear sphericity of *LMNA^WT/WT^* and *LMNA^WT/S143P^* fibroblasts at 37°C, after 1–4 h HS at 44°C, and after 3 h and 24 h recovery at 37°C (*n*=50, from three biological replicates). *P<0.05; **P<0.01; ***P<0.001 (two-way ANOVA with Tukey’s post hoc test). (H) Scatter plot of nuclear sphericity versus nuclear area upon 0–2 h HS at 44°C (*n*=70, two biological replicates). **P<0.01; ***P<0.001 (two-tailed unpaired *t*-test). (I) Scatter plot of nuclear sphericity versus pSer22 lamin A/C intensity upon 0–2 h HS at 44°C (*n*=70). **P<0.01; ***P<0.001 (two-tailed unpaired *t*-test). Boxplots show the 75th, 50th and 25th percentiles, and the whiskers show the 95% c.i. for median. Line plots show the mean±s.e.m. Pearson correlation coefficients (R) are indicated.

As HS at 42°C is within the range of physiological stress that fibroblasts are able to handle (Fig. S2), we wanted to use HS at 44°C to determine how the cells respond to more severe stress when the defense mechanisms are exceeded (Fig. 2A). The baseline pSer22 status was slightly higher in the patient cells compared to control cells (Fig. 2A,B; *P*<0.05). Although the HS-induced increase in lamin A/C phosphorylation followed the same kinetics in both cell lines, the pSer22 levels remained consistently higher in the patient cells than in the control cells (Fig. 2A). Of note, lamin A/C phosphorylation increased several folds higher in cells exposed to severe HS compared to cells subjected to mild HS (Fig. 2A; Fig. S2A). As 4 h HS at 44°C led to irreversible cell injury in most cells, we decided to concentrate on the 1 h and 2 h time points. Following 1 h HS at 44°C, there was a slight but statistically significant increase in the nucleoplasm/lamina intensity ratio of lamin A/C in both the control and patient cells (Fig. 2C,D; *P*<0.01, Fig. S3). Correspondingly, lamin A/C phosphorylation intensity correlated with increased nucleoplasmic localization when analyzed from individual cells at 37°C and upon 1–2 h HS at 44°C (Fig. 2E; Pearson correlation coefficient *R*=0.38 for control and *R*=0.35 for patient cells, *P*<0.01).

Next, we investigated whether the lamin A/C phosphorylation status correlated with HS-induced morphological changes in individual cells. The nuclear area decreased significantly in both cell lines under 44°C HS (Fig. 2F) and inversely correlated with nuclear sphericity (Fig. 2G,H; Pearson correlation coefficient *R*=−0.62 for control and *R*=−0.65 for patients cells, *P*<0.001). Similarly, the lamin A/C phosphorylation intensity and nuclear sphericity correlated with each other in individual heat-shocked cells (Fig. 2I; Pearson correlation coefficient *R*=0.32 for control and *R*=0.43 for patient cells, *P*<0.001). These results suggest that phosphorylation of lamin A/C facilitates nucleoplasmic localization of lamin A/C, which then results in rounding of the nucleus in response to HS.

### Lamin A/C phosphorylation at Ser22 is mediated by several kinases under HS

**Fig. 3.**
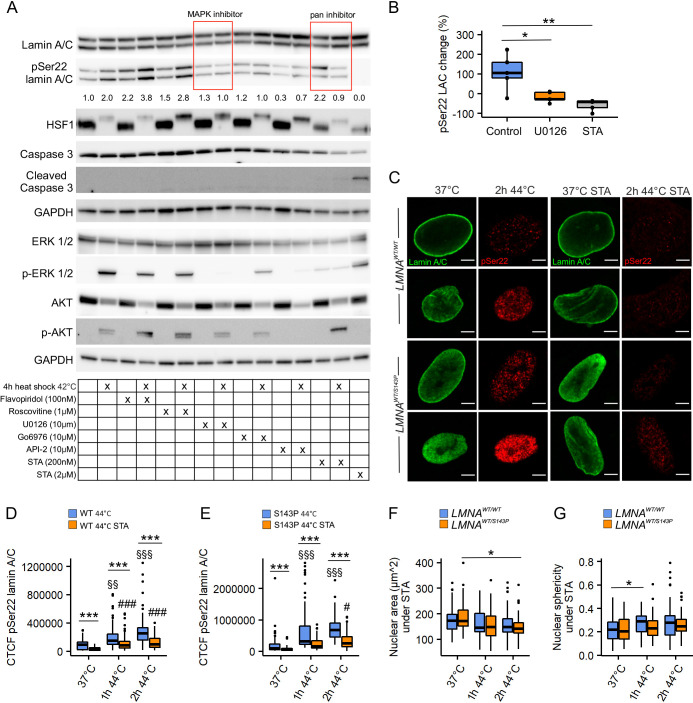
**The effect of kinase inhibition on lamin A/C phosphorylation under HS.** (A) Immunoblots showing lamin A/C, pSer22 lamin A/C, HSF1, ERK1/2, pERK1/2, AKT, pAKT, caspase-3 and cleaved caspase-3 protein levels in control and heat-shocked HeLa cells treated with different kinase inhibitors. GAPDH was used as a loading control and HeLa cells treated with 2 µM staurosporine (STA) as a positive control for apoptotic cell death. The average numerical values of signal intensities relative to the loading control are shown (GAPDH and lamin A/C, *n*=5). (B) Quantification of pSer22 lamin A/C WB intensity change (%) in control cells and cells treated with kinase inhibitors after 4 h HS at 42°C (*n*=5). *P<0.05; **P<0.01 (Mann–Whitney test). (C) Confocal microscopy images of *LMNA^WT/WT^* and *LMNA^WT/S143P^* fibroblasts stained for lamin A/C (green) and pSer22 lamin A/C (red) at normal culture conditions and after 2 h HS at 44°C with or without STA treatment. Scale bars: 5 µm. (D,E) Corrected total cell fluorescence (CTCF) of average pSer22 lamin A/C intensity values under HS and STA treatment (*n*=50, from two biological replicates). ***P<0.001; §, compared to untreated 37°C; #, compared to STA 37°C (Kruskal–Wallis/Dunn’s test). (F,G) Nuclear area (F) and sphericity (G) were determined from *LMNA^WT/WT^* and *LMNA^WT/S143P^* fibroblasts after treatment with STA and exposure to 44°C for 1–2 h (*n*=50, from two biological replicates). *P<0.05 (two-way ANOVA with Tukey’s post hoc test). Boxplots show the 75th, 50th and 25th percentiles, and the whiskers show the 95% c.i. for median.

To identify the potential kinase(s) responsible for lamin A/C phosphorylation during HS, we tested whether the HS response could be prevented in HeLa cells when treated by different kinase inhibitors for 24 h prior to exposure to HS (Fig. 3A). In the presence of specific MAPK (10 μM U0126) and PKC (10 μM Go6976) inhibitors, no increase in lamin A/C phosphorylation was detected upon HS, suggesting that both kinases might be involved in HS-induced lamin phosphorylation. Additionally, MAPK-inhibited cells showed significantly less phosphorylation under HS compared to untreated heat-shocked cells (Fig. 3B, *P*<0.05). Similarly, the generic kinase inhibitor staurosporine (STA, 200 nM; inhibitor of multiple kinases, e.g. MAPK and PKC) reduced lamin phosphorylation compared to unheated cells and heat-shocked control cells (Fig. 3B, *P*<0.05 and *P*<0.01, respectively). AKT inhibition (10 μM API-2) decreased the phosphorylation under normal culture conditions, but lamin A/C phosphorylation was still slightly increased under HS. CDK inhibitors (100 nM flavopiridol and 1 μM roscovitine) had no effect on lamin A/C phosphorylation under HS.

The effect of STA on the nuclear structure of control and patient fibroblasts was further analyzed after 1–2 h HS at 44°C (Fig. 3C). STA treatment significantly reduced the pSer22 lamin A/C labeling intensity in both cell lines, although a minor increase in phosphorylation levels was still detected under HS (Fig. 3D,E). Interestingly, STA treatment appeared to inhibit some morphological changes that were previously detected in heat-shocked cells. Specifically, the area of heat-shocked healthy control cells remained unchanged in the presence of STA, whereas heat-shocked patient cells still showed decreased nuclear area (Fig. 3F; *P*<0.05). In contrast, the nuclear sphericity slightly increased in STA-treated control cells during HS, whereas no significant change was found in the patient cells (Fig. 3G). To conclude, these results suggest that several kinases are directly or indirectly responsible for HS-induced lamin A/C phosphorylation.

### Lamin A/C KO cells exhibit a deformed nuclear shape upon HS

**Fig. 4.**
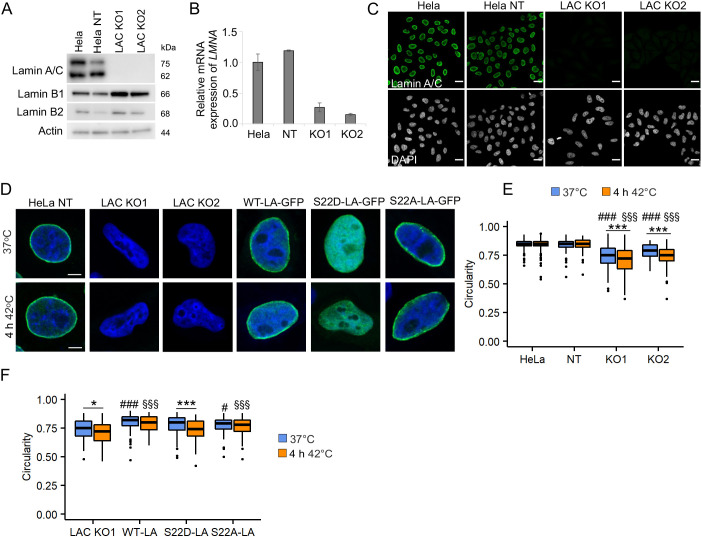
**Knockout of the *LMNA* gene increases nuclear deformability under normal and HS conditions.** (A) Western blot analysis showing the expression of lamin A/C, lamin B1 and lamin B2 in parental HeLa cells and cells treated with non-targeting (NT) sgRNA or two different lamin A/C-targeting sgRNAs (LAC KO1 and LAC KO2). (B) Expression of *LMNA* mRNA in parental HeLa, NT HeLa, LAC KO1 and LAC KO2 cells. (C) Immunofluorescence images of parental HeLa, NT HeLa, LAC KO1 and LAC KO2 cells stained with lamin A/C (green) and DAPI (gray). Scale bars: 20 μm. (D) Representative images from nuclear circularity analysis of NT, KO1 and KO2 cells, as well as KO1 cells transfected with either GFP-tagged WT-LA, S22D-LA or S22A-LA cells at 37°C and after 4 h HS at 42°C. HeLa NT, KO1 and KO2 were stained for lamin A/C (green). All cells were stained with DAPI (blue). Scale bars: 5 μm. (E) Nuclear circularity of parental HeLa, NT HeLa, LAC KO1 and LAC KO2 cells under normal and HS conditions (*n*=200, from four biological replicates), ***P<0.001, #: compared to HeLa and NT HeLa control, §: compared to HeLa and NT HeLa HS. (F) Nuclear circularity of LAC KO1 cells and cells transfected with either GFP-tagged WT-LA, S22D-LA or S22A-LA under control condition and after 4 h heat shock at 42°C (*n*=100, from four biological replicates). Boxplots show the 75th, 50th and 25th percentiles, and the whiskers show the 95% c.i. for median. *P<0.05; ***P<0.001 (two-way ANOVA with Tukey's post hoc test). #, compared to LAC KO1 control; §, compared to LAC KO1 HS.

To further study the role of lamin A/C under HS, we created stable lamin A/C KO HeLa cell lines using CRISPR/Cas9 technology and lentiviral vectors. Cells transduced with two separate short guide RNAs (sgRNAs; designed to target either exon 1 or exon 3 of the *LMNA* gene) showed no detectable amounts of lamin A/C after single-cell sorting, whereas transduction with non-targeting (NT) sgRNA did not affect lamin expression (Fig. 4A–C). The genomic alterations of the *LMNA* gene in the CRISPR/Cas9-edited cells were further verified with amplicon-based next-generation sequencing. In lamin A/C (LAC) KO1 cells, a single nucleotide (nt) insertion (adenine) in exon 3 was detected 4 nt upstream of the protospacer-adjacent motif (PAM) in all the sequencing reads, suggesting identical alterations in both alleles. In LAC KO2 cells, a single nt insertion (cytosine, 5 nt upstream of PAM) and a 20 nt deletion (13 nt upstream and 4 nt downstream of the PAM) in exon 1 were detected in an approximately 1:1 ratio, indicating different alterations in two alleles. No alterations were found in the control HeLa cells treated with NT sgRNA.

There was no significant difference in the nuclear area between the KO, NT and non-transduced parental cells under normal culture conditions (data not shown). However, lamin A/C KO cells showed a more convoluted nuclear shape under normal culture conditions (Fig. 4D; *P*<0.001) and these nuclear deformations became more evident upon HS, whereas control cells retained their circularity (Fig. 4D,E; *P*<0.001). The ectopic expression of wild-type (WT), phosphomimetic (serine to aspartic acid; S22D) and phosphorylation-deficient (serine to alanine; S22A) mutant forms of GFP-labeled lamin A (LA) in LAC KO1 cells rescued symmetrical nuclear shape under normal culture conditions (Fig. 4F; *P*<0.001). However, only WT-LA and S22A-LA protected nuclear morphology under HS (Fig. 4F; *P*<0.001). As S22D-LA was mostly nucleoplasmic (Fig. 4D), this observation indicates that an intact lamina structure is needed to support nuclear shape under HS.

### Lamin A/C phophorylation at Ser22 is not mediated by HSF1

**Fig. 5.**
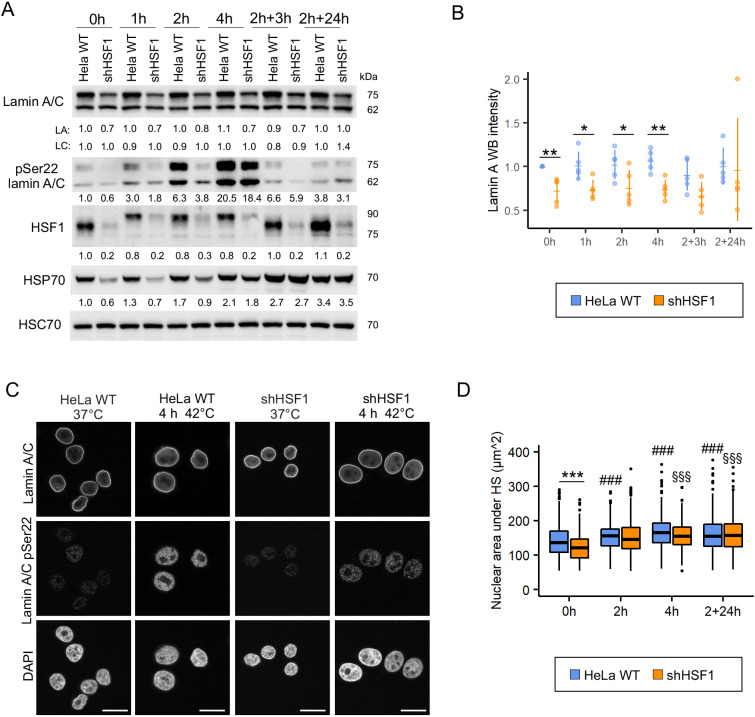
**Lamin A/C phosphorylation at Ser22 is independent of HSF1.** (A) Western blot analysis of lamin A/C, pSer22 lamin A/C, heat shock factor 1 (HSF1), and heat shock protein 70 (HSP70) upon HS and after 3 h and 24 h recovery. The average numerical values of signal intensities relative to the loading control (HSC70) are shown below each blot. pSer22 lamin A/C levels were normalized to HSC70 and lamin A/C levels (*n*=4). (B) Quantification of lamin A WB intensities upon HS and after 3 h and 24 h recovery. Plots show the mean±s.d. *P<0.05; **P<0.01 (Kruskal–Wallis/Dunn’s test). (C) Confocal microscopy images of parental HeLa cells and HSF1-silenced cells stained for lamin A/C, pSer22 lamin A/C and DAPI under normal culture conditions and after 4 h HS. Scale bars: 20 µm. (D) Quantification of nuclear area upon 2–4 h HS and after 24 h recovery. Boxplots show the 75th, 50th and 25th percentiles, and the whiskers show the 95% c.i. for median. ***P<0.001; #, compared to WT control; §, compared to shHSF1 control (two-way ANOVA with Tukey's post hoc test).

As HSF1 is known to influence protein expression during cell stress, we further studied whether HSF1 plays any role in regulating lamin A/C expression and phosphorylation. HeLa cells stably silenced for HSF1 (shHSF1) were exposed to HS for pre-determined periods of time (Fig. 5A). Interestingly, we noticed an overall reduced expression of lamin A but not of lamin C in shHSF1 cells, suggesting that HSF1 might be involved in regulating lamin A protein levels (Fig. 5A,B; *n*=5; *P*<0.05; *P*<0.01). Additionally, phosphorylation of lamin A/C at Ser22 appeared delayed in the shHSF1 HeLa cells upon HS (Fig. 5A), but more detailed analysis showed that pSer22 lamin A/C levels were reduced by 40% in shHSF1 cells under normal conditions (normalized to lamin A/C and HSC70 levels) and the gradual increase in lamin A/C pSer22 levels thereafter was similar to that in HeLa WT cells (Fig. 5A). Immunofluorescence analysis showed no apparent difference in lamin A/C staining in shHSF1 HeLa cells compared to HeLa WT cells (Fig. 5C). However, the nuclear area was significantly smaller in shHSF1 cells compared to HeLa WT cells under basal conditions. This difference might be related to HSF1–JNK–mTORC1 interplay, which has been reported to regulate cell and organ size in mice (Su et al., 2016). Nevertheless, the nuclear area increased under HS and eventually reached the same size in both cell cultures (Fig. 5D).

We also analyzed whether lamin A/C KO affects HSF1 protein expression but found no significant differences in HSF1 expression levels or localization in the KO cells (Fig. S4). In summary, these results suggest that HS-induced phosphorylation of lamin A/C Ser22 is not HSF1 dependent, although the activation and attenuation of HSF1 is closely correlated with the degree of Ser22 phosphorylation. However, lamin A downregulation in shHSF1 cells might indicate an indirect crosstalk between lamin A and HSF1.

### Lap2α is degraded under HS in a lamin A/C-dependent manner

**Fig. 6.**
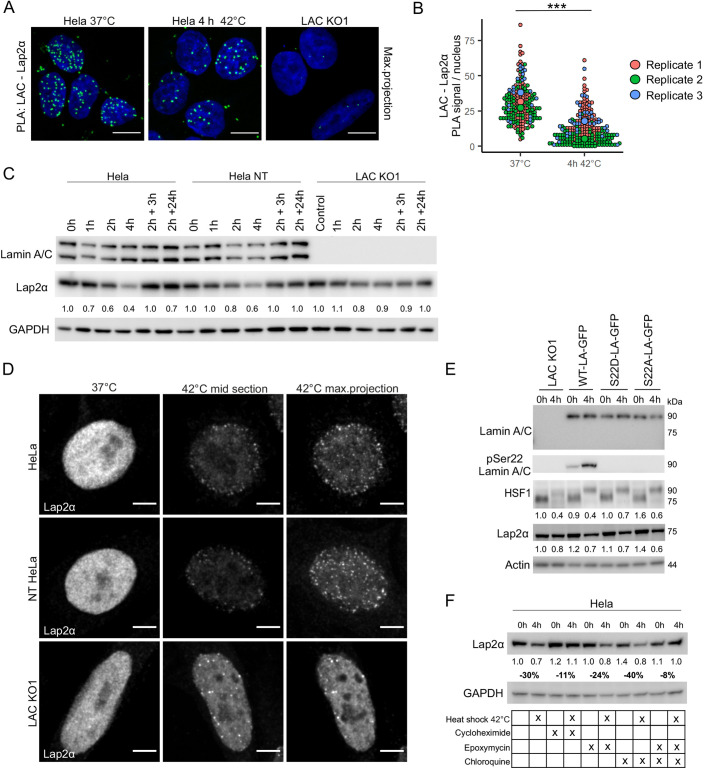
**Lap2α is degraded in a lamin A/C-dependent manner under HS.** (A) Proximity ligation assay (PLA) with antibodies against lamin A/C and Lap2α was carried out on control and heat-shocked cells. Maximum projections of confocal images are shown. Scale bars: 10 μm. Lamin A/C KO1 cells were used as a negative control. (B) Quantification of PLA signals per nucleus (*n*=200, three biological replicates). Data show individual observations and mean values of each replicate. ***P<0.001 (two-tailed unpaired Student's *t*-test). (C) Western blot analysis showing lamin A/C and Lap2α levels in HeLa, NT HeLa and LAC KO1 cells at different time points under HS and at the recovery. The average numerical values of signal intensities relative to the loading control (GAPDH) are shown below the blot. Each cell line has been normalized to its own control sample to highlight the change of Lap2α protein levels between the time points (*n*=5). (D) Confocal microscopy images showing Lap2α aggregation upon 4 h heat shock in HeLa, NT HeLa and LAC KO1 cells. Scale bars: 5 μm. (E) Western blot analysis of LAC KO1 cells transfected with different GFP-tagged lamin A vectors and detected with antibodies against pSer22 lamin A, HSF1 and Lap2α. The average numerical values of signal intensities relative to the loading control (actin) are shown below the blot (*n*=3). (F) Western blot analysis of Lap2α protein levels in heat-shocked parental HeLa cells treated with either 10 μM cycloheximide (CHX), 10 μM epoxymycin (EPO), 10 μM chloroquine (CQ) or both EPO and CQ. The average numerical values of signal intensities relative to the loading control (GAPDH) and percentage of intensity change between control and heat-shocked cells are shown below the blot (*n*=2).

We next asked whether lamin A/C phosphorylation affects its interaction with known binding partners. As the phosphorylation mostly appears in the nucleoplasmic pool of lamin A/C rather than in the lamina region (Fig. 1D), we decided to analyze the crosstalk between lamin A/C and its binding partner Lap2α. Based on proximity ligation assay (PLA), close proximity signals of Lap2α and lamin A/C were reduced in HeLa cells after 4 h HS at 42°C compared to control cells (Fig. 6A,B; *P*<0.001). As the decreased number of PLA signals might be due to reduced proximity or downregulation of either protein, we further tested the abundance of lamin A/C and Lap2α during HS. Although lamin A/C protein levels remained relatively stable, there was a clear downregulation of Lap2α upon HS in parental HeLa and NT HeLa cells, but interestingly not in lamin A/C KO1 or KO2 cells (Fig. 6C; Fig. S5A–D, *P*<0.01). All the heat-shocked cell lines showed distinct Lap2α aggregates, which mostly appeared in the nuclear periphery (Fig. 6D). However, the Lap2α aggregates in the KO cells were approximately 300 nm closer to the center of the nucleus compared to control cells, suggesting that loss of lamin A/C affected their localization (Fig. S6B,C,E). The HS-induced Lap2α downregulation was restored with ectopic expression of either WT-LA, S22D-LA or S22A-LA into lamin A/C KO1 cells (Fig. 6E; Fig. S5E). Additionally, the localization of Lap2α aggregates towards the nuclear periphery was rescued with all the plasmids (Fig. S6D,F).

As HS-induced Lap2α downregulation could be due to reduced protein synthesis or degradation of the protein, we next exposed HeLa cells to HS in the presence of 10 μM cycloheximide (CHX, an inhibitor of protein synthesis), 10 μM epoximycin (EPO, a proteosomal inhibitor) and/or 10 μM chloroquine (CQ, an inhibitor of autophagy) (Fig. 6F). Surprisingly, Lap2α was not downregulated or aggregated in heat-shocked cells under CHX treatment. As CHX inhibits all protein synthesis, it is possible that the proteins required for Lap2α degradation were not produced. This also indicates that Lap2α downregulation is due to degradation of the protein rather than inhibition of protein synthesis. Lap2α was equally downregulated upon HS in the presence of EPO or CQ. However, in the presence of both EPO and CQ, Lap2α protein levels remained stable. These results suggest that under prolonged HS, Lap2α is degraded possibly by both proteasomes and autophagy, and the degradation is impaired in lamin A/C KO cells.

### Dermal patient fibroblasts with a *LMNA* mutation are more sensitive to HS

**Fig. 7.**
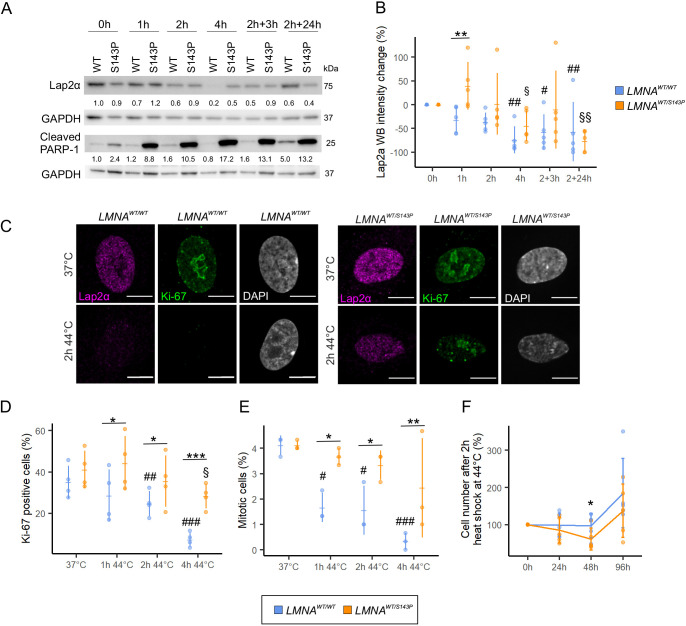
***LMNA* mutant cells are hypersensitive to severe HS.** (A) Western blot analysis of *LMNA^WT/WT^* and *LMNA^WT/S143P^* fibroblasts as detected with antibodies against Lap2α and cleaved PARP-1. The average numerical values of signal intensities relative to the loading control (GAPDH) are shown below each blot (Lap2α, *n*=5; cleaved PARP-1, *n*=2). (B) Quantification of Lap2α WB intensities under HS (*n*=5). (C) Representative confocal images of *LMNA^WT/WT^* and *LMNA^WT/S143P^* cells cultured in normal conditions or exposed to 44°C for 2 h prior to fixation. The cells were stained for Lap2α (magenta), Ki-67 (green) and DAPI (gray). Scale bars: 10 μm. (D) The percentage of Ki-67-positive cells at 37°C and after 1–4 h at 44°C (*n*=1200, from four biological replicates). (E) The percentage of mitotic cells at 37°C and after 1–4 h HS at 44°C (*n*=900, from three biological replicates). (F) Cell numbers counted after 2 h HS at 44°C (time point 0 h) and after 24 h, 48 h and 96 h recovery at 37°C (*n*=5). Line plots show the mean±s.d. and datapoints from each replicate. *P<0.05; **P<0.01; ***P<0.001 (Kruskal–Wallis/Dunn’s test). #, compared to *LMNA^WT/WT^* control; §, compared to *LMNA^WT/S143P^* control.

To study whether the changes in Lap2α also take place in normal diploid cells, control and patient fibroblasts with a p.S143P *LMNA* mutation were tested. Similar to HeLa cells, Lap2α was downregulated in heat-shocked control fibroblasts (Fig. 7A,B; *P*<0.05). However, Lap2α downregulation was delayed in the patient cells and evident only after 4 h HS and at the recovery (Fig. 7A,B; *P*<0.05). Further analysis revealed that Lap2α was downregulated in parallel with reduced expression of the proliferation marker Ki-67 (Fig. 7C). In control cells, the number of Ki-67-positive cells decreased from 35% to 7% during 4 h HS, whereas in the patient cells, a significantly smaller reduction from 41% to 28% was observed (Fig. 7D; *P*<0.001 and *P*<0.05, respectively). Correspondingly, there were significantly more mitotic cells among patient cells compared to controls during HS (Fig. 7E, *P*<0.05). These results suggest that HS-induced cell cycle arrest to G0 is impaired in lamin A/C mutant cells. Additionally, cleaved poly (ADP-ribose) polymerase-1 (PARP-1) levels were significantly higher in the patient cells throughout the experiment, potentially indicating caspase activity and increased DNA damage in these cells (Fig. 7A). Cell viability was further analyzed with a cell count assay. HS at 44°C reduced the survival of patient cells (presumably due to increased cell death) within the first 48 h of recovery (Fig. 7F; *P*<0.05). Both the cell lines eventually recovered from the HS and started to proliferate after 48 h. Altogether, these results suggest that patient cells have a decreased capacity to undergo cell cycle arrest, which might eventually lead to reduced cell survival at the recovery.

## DISCUSSION

In the current study, we describe a previously unreported and evolutionarily conserved post-translational modification of the lamin A/C amino-terminal head upon HS. Lamin A/C was gradually phosphorylated at Ser22 in both normal diploid and transformed human and mouse cell lines, as well as in *D. melanogaster* flies *in vivo.* Consequently, the phosphorylated pool of lamin A/C relocated into the nucleoplasm, and the adapted nuclei became more spherical, especially under severe HS. However, lamin A/C KO and mutant cells were more vulnerable to HS, indicating that normal filamentous lamina, formed by unphosphorylated lamins, is equally important for nuclear shape maintenance and regulation of the cell cycle through Lap2α in heat-shocked cells (Fig. S7). All these results suggest that the nuclear lamina is dynamically modified under HS to preserve cell homeostasis.

### The effects of HS-induced phosphorylation on lamin A/C

In early studies, Krachmarov and Traub (1993) reported heat-induced stabilization of the nucleoskeleton and dephosphorylation of lamin A/C in Ehrlich ascites tumor cells after HS. Additionally, Smith et al. (1987) reported that in *Drosophila* Schneider 2 cells, lamin Dm2 is dephosphorylated to lamin Dm1 under HS. In contrast to these studies describing the overall phosphorylation state of lamins, we focused on specific lamin A/C phospho-epitopes. Thus, our results do not necessarily contradict with the previous observations, although we found no clear evidence for general dephosphorylation of lamin A/C upon HS by LC-MS/MS analysis.

Ser22 is a canonical mitotic phosphorylation site, but accumulating data show that Ser22 is also phosphorylated in interphase cells upon low mechanical stress and on soft matrices, leading to increased elasticity of the nuclear lamina and improved cellular adaption (Bainer and Weaver, 2013; Buxboim et al., 2014). Our results align with the literature as we demonstrated that lamin A/C phosphorylation increases the nucleoplasmic localization of lamin A/C and simultaneously causes a more spherical nuclear shape in response to HS (Fig. 2C–I). Hence, the pSer22 response causes a partial change in lamin A/C phosphorylation stoichiometry, which is insufficient to induce major disassembly, but rather induces a partial shift from the assembled to the nucleoplasmic lamin pool. Such finetuning is likely to serve the regulation of nuclear structure and properties that facilitate nuclear adaptation during ongoing stressful conditions.

The increased nuclear sphericity during HS is presumably a consequence of cell shrinking. Gungor et al. (2014) reported a drastic reduction of F-actin expression and cell rounding or shrinking in response to severe heat treatment (43°C). The resultant reduced cytoskeletal tension also decreases tension on the nucleus and facilitates its rounding through lamin A/C phosphorylation (Buxboim et al., 2014). In accordance with the assumption that increased nuclear sphericity is due to phosphorylation-mediated effects, we found that the nuclear sphericity of fibroblasts was diminished when lamin A/C phosphorylation was inhibited (Fig. 3G).

More recently, it was reported that in interphase cells, pSer22 lamin A/C might act as a transcriptional activator by binding gene enhancer domains in the nuclear interior, and some of the binding sites were altered in dermal fibroblasts obtained from HGPS patients (Ikegami et al., 2020). As the transcriptional response to HS results in the repression of thousands of genes (Mahat et al., 2016), it is tempting to speculate that phosphorylated lamin A/C contributes to HS-induced gene expression, similar to HSF1, and further studies on these mechanisms are warranted.

We also noticed that lamin A/C was constantly more phosphorylated at Ser22 in the patient fibroblasts compared to control cells (Fig. 2A,B). As p.S143P mutant lamin A/C is incapable of forming normal filaments and is primarily nucleoplasmic (West et al., 2016), it might be more accessible for phosphorylation by kinases in these cells. Ser22 phosphorylation is known to increase the mobility of lamin A (Kochin et al., 2014), and thus the increased phosphorylation of lamin A/C might further reduce the stability of lamina in the patient cells.

### Multiple kinases might phosphorylate lamin A/C under HS

To determine which kinases were responsible for the HS-induced phosphorylation of lamin A/C on Ser22, we inhibited several different kinases previously shown to phosphorylate lamin A/C (reviewed by Liu and Ikegami, 2020). MAPK and PKC inhibitors partially abolished lamin A/C phosphorylation, whereas the pan-inhibitor STA had the most significant effect, suggesting that several kinases might be involved (Fig. 3A). Our results are consistent with previous screening studies showing that lamin A and C are ERK2 substrates (Carlson et al., 2011). There is also evidence that lamin A is phosphorylated at Ser268 by PKC to regulate nuclear size (Edens et al., 2017). Interestingly, we also detected the phosphorylation of Ser268 in LC-MS/MS analysis after 2[Supplementary-material sup1]h and 4[Supplementary-material sup1]h HS but not in control cells (Table S1). Therefore, phosphorylation of lamin A/C at Ser268 might contribute to the modulation of nuclear size upon HS. As at least 25 residues in lamin A/C are known to be phosphorylated during interphase and the degree of phosphorylation at each site might vary (Liu and Ikegami, 2020), one still needs to be cautious when estimating the biological effect of phosphorylation on a single residue.

### The effects of Lap2α degradation on cell survival upon HS

Our results also revealed that Lap2α is degraded under HS, and the degradation is impaired in lamin A/C KO cells (Fig. 6C; Fig. S5A,B). Transfections with WT-LA, S22D-LA and S22A-LA restored the degradation of Lap2α (Fig. 6E), which suggest that Lap2α is degraded in a lamin A/C-dependent manner but is independent of the lamin A/C Ser22 phosphorylation status. A similar downregulation was detected in primary human skin fibroblasts derived from healthy control and a dilated cardiomyopathy patient carrying the p.S143P *LMNA* mutation (Fig. 7A). We also noticed that Lap2α is degraded simultaneously with the reduction of Ki-67 levels in control fibroblasts under severe HS, but this degradation appeared delayed in the *LMNA* mutant patient fibroblasts (Fig. 7B,C). Previous data have shown that Lap2α is required for cell proliferation and is degraded upon cell cycle arrest in normal human fibroblasts (Pekovic et al., 2007). Based on these results, we conclude that lamin mutant fibroblasts have a decreased capacity to undergo cell cycle arrest to G0 under HS. Similar results have been reported in *Lmna^−/−^* mouse embryonic fibroblasts, which showed diminished cell cycle arrest in response to γ-irradiation-induced DNA damage (Johnson et al., 2004). A continued cell cycle under stressful conditions typically leads to accumulating DNA damage and eventually cell death through apoptosis. Accordingly, PARP-1 was increasingly cleaved in lamin mutant patient cells under 44°C HS, and the cells showed reduced survival at recovery compared to control cells (Fig. 7F). In support of these findings, hypersensitivity to HS has previously been reported in dermal fibroblasts obtained from HGPS patients carrying the G608G mutation in *LMNA* gene. These cells showed dysmorphic nuclei and delayed recovery after 30 min HS at 45°C (Paradisi et al., 2005). Extensive nuclear deformations were also detected in heat-shocked dermal fibroblasts from FPLD patients carrying the R482Q mutation in the *LMNA* gene (Vigouroux et al., 2001). Taken together these results indicate that in heat-shocked cells, in which the lamina is rapidly remodeled through phosphorylation, the disease-linked mutations in lamin A/C cause nuclear instability and might sensitize cells to cell death.

In conclusion, we discovered a previously unknown and evolutionarily conserved mechanism that regulates lamin A/C dynamics in heat-shocked cells. The degree of lamin A/C Ser22 phosphorylation appears tightly regulated and correlates with the duration and severity of HS, suggesting that the ratio of phosphorylated and unphosphorylated lamin A/C is critical for the maintenance of proper lamina elasticity and/or stiffness, depending on the prevailing circumstances. Our results also show that HS-induced modifications in the lamina are very similar to those induced by mechanical cues, such as matrix elasticity (Buxboim et al., 2014). It seems plausible that HS-induced cellular shrinking facilitates Ser22 phosphorylation, which in turn increases nuclear sphericity upon HS. Which kinases are specifically responsible for Ser22 phosphorylation and whether pSer22 lamin A/C also plays a role in HS-induced gene expression remains to be elucidated in follow-up studies.

## MATERIALS AND METHODS

### Cell lines, cell culture and transfections

HeLa cervical carcinoma cells were grown under a humidified 5% CO_2_ atmosphere at 37°C in Dulbecco's modified Eagle medium (DMEM, Lonza, Alpharetta, GA) supplemented with 10% fetal bovine serum (FBS; Thermo Fisher Scientific, Waltham, MA) and penicillin/streptomycin/glutamine (Thermo Fisher Scientific). HSF1-silenced HeLa cells (shHSF1) were kindly provided by Prof. Lea Sistonen (Åbo Akademi University, Turku, Finland). Primary human and mouse fibroblasts were cultured in Minimum Essential Media (MEM, Thermo Fisher Scientific) supplemented with 15% FBS, antibiotics (penicillin and streptomycin) and 5% non-essential amino acids (Thermo Fisher Scientific). The use of patient fibroblasts was approved by the Ethics Committees of the Hospital District of Helsinki and Uusimaa (HUS 387/13/03/2009 and HUS/1187/2019). All procedures were undertaken with informed consent and according to the principles expressed in the Declaration of Helsinki.

HeLa cells and mouse fibroblasts were heat shocked under a humidified 5% CO_2_ atmosphere at 42°C for 1, 2 or 4 h and left to recover at 37°C for 3 or 24 h after 2[Supplementary-material sup1]h HS. Human fibroblasts were heat shocked either at 42 or 44°C. For cell cycle synchronization, 10 μg/ml aphidicolin (Sigma-Aldrich, St. Louis, MO) was used 24 h prior to HS. For kinase inhibition, 100 nM flavopiridol, 1 μM roscovitine, 10 μM U0126, 10 μM Go6976, 10 μM triciribine (API-2) or 200 nM staurosporine (STA) (all purchased from Selleckchem, Houston, TX) was used 24 h prior to exposure to HS. For degradation analysis, we exposed HeLa cells to HS in the presence of 10 μM cycloheximide (CHX, Sigma-Aldrich), 10 μM epoximycin (EPO, Sigma-Aldrich), and 10 μM chloroquine (CQ, Sigma-Aldrich). For control cells, equal concentrations of solvent (DMSO) were added into culture media.

Lamin A/C KO HeLa cells were transfected with Trans-IT HelaMONSTER (MirusBio, Madison, WI) according to the manufacturer's protocol and used for experiments 48 h after the transfection. Plasmids encoding either GFP-tagged WT lamin A (pGFP-C1-LA) and phosphomimetic (pGFP-C1-LA-S22D) or phospho-deficient (pGFP-C1-LA-S22A) mutant forms of lamin A were cloned as described elsewhere (Kochin et al., 2014).

The human fibroblast count was determined after 2 h HS (44°C) at 0, 24, 48 and 96 h time points. The attached cells were trypsinized at each time point, collected and counted with cell counting slides (Bio-Rad, Hercules, CA).

### Genome editing with CRISPR/Cas9

Lamin A/C KO HeLa cells were established with CRISPR/Cas9 technology at the Turku Bioscience Genome Editing Core using a two-component CRISPR system (Adli, 2018). The *LMNA* sgRNAs (seq#1, 5′-CCAGAAGAACATCTACAGTG-3′; seq#2, 5′-TGAAGAGGTGGTCAGCCGCG-3′; and seq#3, 5′-ATGCCAGGCAGTCTGCTGAG-3′) were selected using the DeskGEN platform (Desktop Genetics; software no longer available) and cloned according to Feng Zhang laboratory protocol (https://media.addgene.org/data/plasmids/52/52963/52963-attachment_IPB7ZL_hJcbm.pdf; Sanjana et al., 2014).

Separate lentivectors containing spCas9 (lentiCas9-Blast, Addgene #52962) and sgRNA (lentiGuide-Puro, Addgene #52963) (both deposited by Feng Zhang) were produced in the HEK 293FT packaging cell line by transient co-transfection. Shortly, 40–70% confluent HEK 293FT cells were used for transfections with 14 μg of the transfer vector, 4 μg of the packaging vector psPAX2 (Addgene #12260, deposited by Didier Trono) and 2 μg of the envelope vector pMD2.G (Addgene #12259) mixed in 0.45 ml water, 2.5 M CaCl_2_ and 2× HeBS (274 mM NaCl, 10 mM KCl, 1.4 mM Na_2_HPO_4_, 15 mM D-glucose, 42 mM HEPES, pH 7.06) per 10 cm dish. Before adding to the cells, the DNA-HeBS mix was incubated for 30 min at room temperature. After overnight incubation, the medium with DNA precipitate was gently removed from the cells and replaced with a full fresh medium. Media containing viral particles were collected after 72 h, spun at 500 ***g*** for 5 min at room temperature to remove cell debris, filtered through a 0.45 µm PES syringe filter (Fisherbrand, Thermo Fisher Scientific), and concentrated by ultracentrifugation for 2 h at 107 000 ***g*** (maximum) at 4°C (Beckman Coulter, SW 32Ti rotor). The pellet containing lentiviral particles was suspended in the residual medium, incubated for ∼2 h at 4°C with occasional mild vortexing, aliquoted, snap frozen and stored in −70°C. A QuickTiter Lentivirus-Associated p24 ELISA assay (Cell Biolabs) was used to measure the physical lentiviral titer with a serial dilution of virus stock, according to the manufacturer’s protocol.

To generate lamin A/C KO HeLa cells, ∼100,000 cells were seeded on a 24-well plate. A day later, the cells were transduced with Lenti-Cas9 (multiplicity of infection of 1, 3 and 6) and, 72 h later, 8 μg/ml of blasticidin S HCl (Thermo Fisher Scientific) was applied to select Cas9-expressing cells. Cells transduced with the smallest amount of Lenti-Cas9 particles that survived after the control well were used for the next step. In the second stage, the mixed pool of stably expressing Cas9 cells was transduced with Lenti sgRNA vectors (multiplicity of infection of 6, 9 and 12) and, 72 h later, 1 μg/ul puromycin (Thermo Fisher Scientific) was applied on cells to select double-positive Cas9^+^/Lenti sgRNA^+^ cells. Based on western blotting results, cell populations showing the highest reduction in lamin A/C protein levels were single sorted (SH800 cell sorter, Sony Biotechnology) and re-grown into a clonal cell population. On average, 20 clones per sgRNA population were screened with western blotting. Both sgRNA#1 (designed to target *LMNA* exon 3) and sgRNA#2 (designed to target *LMNA* exon 1) generated full lamin A/C KO clones that were chosen for further analyses, and named LAC KO1 and LAC KO2, respectively.

The genomic alterations of the *LMNA* gene in CRISPR/Cas9-edited cells were verified with amplicon-based next-generation sequencing using the MiSeq Illumina platform at the DNA Sequencing and Genomics Laboratory (BIDGEN, University of Helsinki, Finland). In brief, genomic DNA was extracted from HeLa LAC KO1 and LAC KO2 cells, as well as from control HeLa cells treated with a NT sgRNA, using an ISOLATE II Genomic DNA kit (Meridian Bioscience, Cincinnati, OH). Genomic regions containing the sgRNA-targeting sites were amplified using KAPA HIFI HS ReadyMix (NIPPON Genetics, Tokyo, Japan) with specific primers (5′-TTCAGGATGAGATGCTGCGG-3′ and 5′-CAATCTCCACCAGTCGGGTC-3′ for LAC KO1 cells; 5′-AATGATCGCTTGGCGGTCTA-3′ and 5′-CAATTCCCCTTGACACTGCC-3′ for LAC KO2 cells) containing the following overhang sequences: 5′-ACACTCTTTCCCTACACGACGCTCTTCCGATCT-3′ and 5'-GTGACTGGAGTTCAGACGTGTGCTCTTCCGATCT-3′. PCR reactions included denaturation at 95°C for 3 min, followed by 20 cycles at 95°C for 30 s, at 60°C for 1 min and at 72°C for 1 min. PCR amplicons were treated with exonuclease I and shrimp alkaline phosphatase for 30 min at 37°C to remove excess free primers. One microliter of the PCR product was used for dual-indexing PCR with denaturation at 98°C for 30 s, followed by 14 cycles at 98°C for 10 s, at 65°C for 30 s and at 72°C for 10 s using Phusion Hot-Start II DNA Polymerase (Thermo Fisher Scientific) and indexing primers (BIDGEN's in-house design) that have unique combinations of 8 bp i5 and i7 indexes. PCR products were finally pooled and purified with MagSi-NGS plus beads (Magtivio, Nuth, Netherlands) at a ratio of 0.9×. The library pool was sequenced on the MiSeq Illumina platform with paired-end reads (326 bp for read 1 and 278 bp for read 2) at a final library concentration of 9.5 pM using a MiSeq V3 600 cycle flow cell (Illumina). After sequencing, the overhangs were trimmed by Cutadapt software (Martin, 2011), sequences mapped onto the reference sequence Human_reference_sequence_GRCh38.p13 by Burrows–Wheeler Aligner software (Li and Durbin, 2009) and visualized by Integrative Genome Viewer (Robinson et al., 2011).

### *D. melanogaster* husbandry, treatment and analysis

Canton-S WT flies were a gift from Dr Pascal Meier, Institute of Cancer Research, London, UK. Flies were maintained in vials at 22°C on a 12 h/12 h light/dark cycle on Nutri-Fly cornmeal medium (Nutri-fly BF, Dutscher Scientific, France). Adult flies were used in the experiments. HS was induced in the flies by incubating the vials (20–30 flies per vial) at 37°C for 30 min, followed by 3 or 7 h recovery periods at 22°C. Ten flies per sample were kept at −20°C for 5 min to euthanize the flies. Using an electronic pestle, the flies were homogenized in lysis buffer (50 mM Tris-HCl pH 7.5, 150 mM NaCl, 1% Triton X-100, 1 mM EDTA, 10% glycerol) with 1× protease and phosphatase inhibitors (Thermo Fisher Scientific). The lysates were kept on ice for 10 min. To remove exoskeleton and fat from the lysates, the samples were centrifuged for 10 min at 13,800 ***g*** at 4°C. The clear supernatant was transferred into new microtubes, avoiding the debris at the bottom and the fat layer on the top. The lysate was cleared by centrifuging again for 10 min at 13,800 ***g*** at 4°C. The clear supernatant was transferred into new microtubes and mixed with 4× Laemmli β-mercaptoethanol to a concentration of 1×. Samples were incubated at 95°C for 10 min, separated on 10% SDS-PAGE gels and transferred to a nitrocellulose membrane. *Drosophila* lamin C and phosphorylated lamin C were detected with mouse anti-lamin C antibody (1:1000, LC28.26, Developmental Studies Hybridoma Bank, Iowa City, IA) and rabbit monoclonal phospho-lamin A/C (Ser22) (1:1000, D2B2E, Cell Signaling Technology, Danvers, MA), respectively. Actin was used as a loading control and was detected with goat polyclonal actin antibody (1:2000, C11, sc-1615, Santa Cruz Biotechnology, Dallas, TX). The secondary antibodies were goat anti-mouse IgG-HRP (sc-2005), goat anti-rabbit IgG-HRP (sc-2004) and donkey anti-goat IgG-HRP (sc-2020) (all from Santa Cruz Biotechnology) at 1:5000. The ECL advanced western blotting detection kit (RPN 3243, Sigma-Aldrich) was used for signal detection.

### Immunoblotting

Unheated and heated samples were lysed in mammalian protein extraction reagent (M-PER, Thermo Fisher Scientific) complemented with 1× protease and 1× phosphatase inhibitors (Thermo Fisher Scientific). Cell lysates were mixed with 4× Laemmli sample buffer, run on a 4–20% gradient gel (Bio-Rad) and transferred to nitrocellulose filter (Bio-Rad). The primary antibodies used were: mouse monoclonal anti-lamin A/C (1:10,000, 5G4, kindly provided by Prof. Robert D. Goldman, Northwestern University, IL), anti-lamin B1 (1:1000, ab16048, Abcam) rabbit polyclonal anti-heat shock factor 1 (1:1000, ADI-SPA-901, Enzo Life Sciences, Farmingdale, NY), rat monoclonal anti-heat shock factor 1 (1:1000, 10H8, StressMarq Biosciences, Victoria, Canada), rabbit monoclonal anti-phospho-lamin A/C serine 22 (1:1000, D2B2E, Cell Signaling Technology), rabbit polyclonal anti-phospho-lamin A/C serine 392 (1:1000, ab58528, Abcam), rabbit polyclonal anti-AKT (pan) (1:1000, C67E7, Cell Signaling Technology), rabbit monoclonal anti-pAKT T303 (1:1000, D6F8, Cell Signaling Technology), rabbit polyclonal anti-ERK1 (1:500; K-23, Santa Cruz Biotechnology), rabbit polyclonal anti-ERK2 (1:500, c-14, Santa Cruz Biotechnology), rabbit monoclonal anti-pERK1/2 (1:1000, D13.14.4E, Cell Signaling Technology), rabbit monoclonal anti-cleaved PARP-1 (1:1000, E-51, Abcam), rabbit monoclonal anti-caspase-3 (1:1000, 8G10, Cell Signaling Technology), rabbit monoclonal anti-cleaved caspase-3 (1:1000, Asp175, Cell Signaling Technology), rabbit monoclonal anti-Lap2α (1:5000, 245/2, kindly provided by Prof. Roland Foisner, University of Vienna), mouse monoclonal anti-HSP70/HSP72 (1:1000, C92F3A-5, Enzo Life Sciences), rat monoclonal anti-HSC70/HSP73 (1:1000, 1B5, Enzo Life Sciences), HRP-conjugated anti-GAPDH (1:5000, ab9385, Abcam) and mouse monoclonal anti-actin (1:1000, AC-40, Sigma-Aldrich). The secondary antibodies were HRP-conjugated donkey anti-rabbit-IgG, sheep anti-mouse-IgG and anti-rat-IgG (all from GE Healthcare, Chicago, IL). The antibodies were detected with Enhanced Chemiluminescence kit (Thermo Fisher Scientific).

### Immunofluorescence and microscopy

Cells grown on coverslips were fixed in 10% formalin for 10 min, permeabilized with 0.1% Triton X-100 for 10 min and blocked with 1% bovine serum albumin in TBS containing 0.1% Tween 20 for 30 min. The primary antibodies used were mouse monoclonal anti-lamin A/C (1:10,000, 5G4, kindly provided by Prof. Robert D. Goldman, Northwestern University, IL), mouse monoclonal anti-lamin A/C (1:100, 4C11, Cell Signaling Technology), rabbit monoclonal anti-phospho-lamin A/C serine 22 (1:400, D2B2E, Cell Signaling Technology), rabbit polyclonal anti-HSF1 (1:400, ADI-SPA-901, Enzo Life Sciences), rat monoclonal anti-HSF1 (1:400, 10H8, StressMarq Biosciences), rabbit monoclonal anti-Lap2α (1:1000, 245/2, kindly provided by Prof. Roland Foisner, University of Vienna) and mouse monoclonal Ki-67 (1:200, MIB-1, DAKO, Denmark). The secondary antibodies were donkey anti-rabbit IgG conjugated to Alexa Fluor 488, donkey anti-mouse IgG conjugated to Alexa Fluor 555, and chicken anti-rat IgG conjugated to Alexa Fluor 647 (all Molecular Probes, 1:200, Eugene, OR). ProLong Diamond Antifade Mountant with DAPI was used to visualize DNA (Thermo Fisher Scientific). Dualink proximity ligation assay (PLA) was conducted according to the manufacturer's protocol (Sigma-Aldrich).

The spinning-disk confocal microscope used was a 3i Marianas with Yokogawa CSU-W1 scanning unit on an inverted Zeiss AxioObserver Z1 microscope, controlled by SlideBook 6 software (Intelligent Imaging Innovations, Göttingen, Germany). The objective used was 63×/1.4 oil. Images were acquired with ORCA Flash4 sCMOS camera (Hamamatsu Photonics, Hamamatsu, Japan). All the images were analyzed with ImageJ Fiji software (Schindelin et al., 2012). Confocal maximum-projection images were used to analyze the quantity of PLA signals. Lap2α aggregate quantity and localization were analyzed from 3D confocal stacks using the ImageJ plugin NucleusJ (Poulet et al., 2015). Fluorescence intensities of lamin A/C within the lamina (L) and nucleoplasmic (N) regions were quantified and the ratio of fluorescence between the lamina and nucleoplasma was calculated as follows:

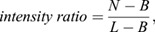
where B is the background fluorescence.

The shHSF1 samples were imaged using a Zeiss LSM880 confocal microscope with Airyscan mode. Images were taken using a 63× Zeiss C plan-Apochromat oil immersion objective (NA=1.4) with an additional 1.8× zoom. Following imaging, the raw data were processed using the Airyscan processing module in the Zen blue software (Jena, Germany). Minor image quality adjustments were done using the ImageJ FIJI software.

### Phosphorylation analysis by mass spectrometry

Lamin A/C was immunoprecipitated from G1/S phase-synchronized control and heat shocked (2 and 4 h at 42°C) HeLa cells and run on gels. Mass spectrometry analysis was performed at the Turku Proteomics Facility, University of Turku and Åbo Akademi University with the following protocol: the lamin A/C immunoprecipitate was in-gel digested using trypsin. Phosphopeptides were enriched by the High-Select TiO2 Phosphopeptide Enrichment Kit (Thermo Fisher Scientific) according to the manufacturer's protocol. The LC-ESI-MS/MS analyses were performed on a nanoflow HPLC system (Easy-nLC1200, Thermo Fisher Scientific) coupled to the Q Exactive HF mass spectrometer (Thermo Fisher Scientific) equipped with a nano-electrospray ionization source. MS data were acquired automatically by using Xcalibur 3.1 software (Thermo Fisher Scientific). Data files were searched for protein identification using Proteome Discoverer 2.3 software (Thermo Fisher Scientific) connected to an in-house server running the Mascot 2.6.1 software (Matrix Science). Data were searched against the SwissProt database containing human protein sequences.

### Statistical analysis

Each experiment was repeated at least two times from samples that were prepared independently of each other. The statistical analyses were performed using GraphPad Prism 8 or R software. Datasets that followed normal distribution were analyzed with two-tailed unpaired Student's *t*-test or grouped two-way ANOVA followed by Tukey’s post hoc test. Pearson correlation coefficient was used to quantify the relationship between two variables. Non-parametric datasets were analyzed with Mann–Whitney test or Kruskal–Wallis test followed by Dunn’s test for multiple comparisons. *P*-values ≤0.05 were considered statistically significant and are marked with symbols in the figures. The exact sample sizes and number of measurements for each experiment are indicated in the figure legends.

## Supplementary Material

10.1242/joces.259788_sup1 Supplementary information
