# Supplementary material for: Lamin A/C phosphorylation at serine 22 is a conserved heat shock response to regulate nuclear adaptation during stress
Source: J Cell Sci. 2023 Feb 27;136(4):jcs259788. doi: 10.1242/jcs.259788 (PMC10022683; doi:10.1242/jcs.259788)
Supplement: Supplementary information [file joces-136-259788-s1.pdf]

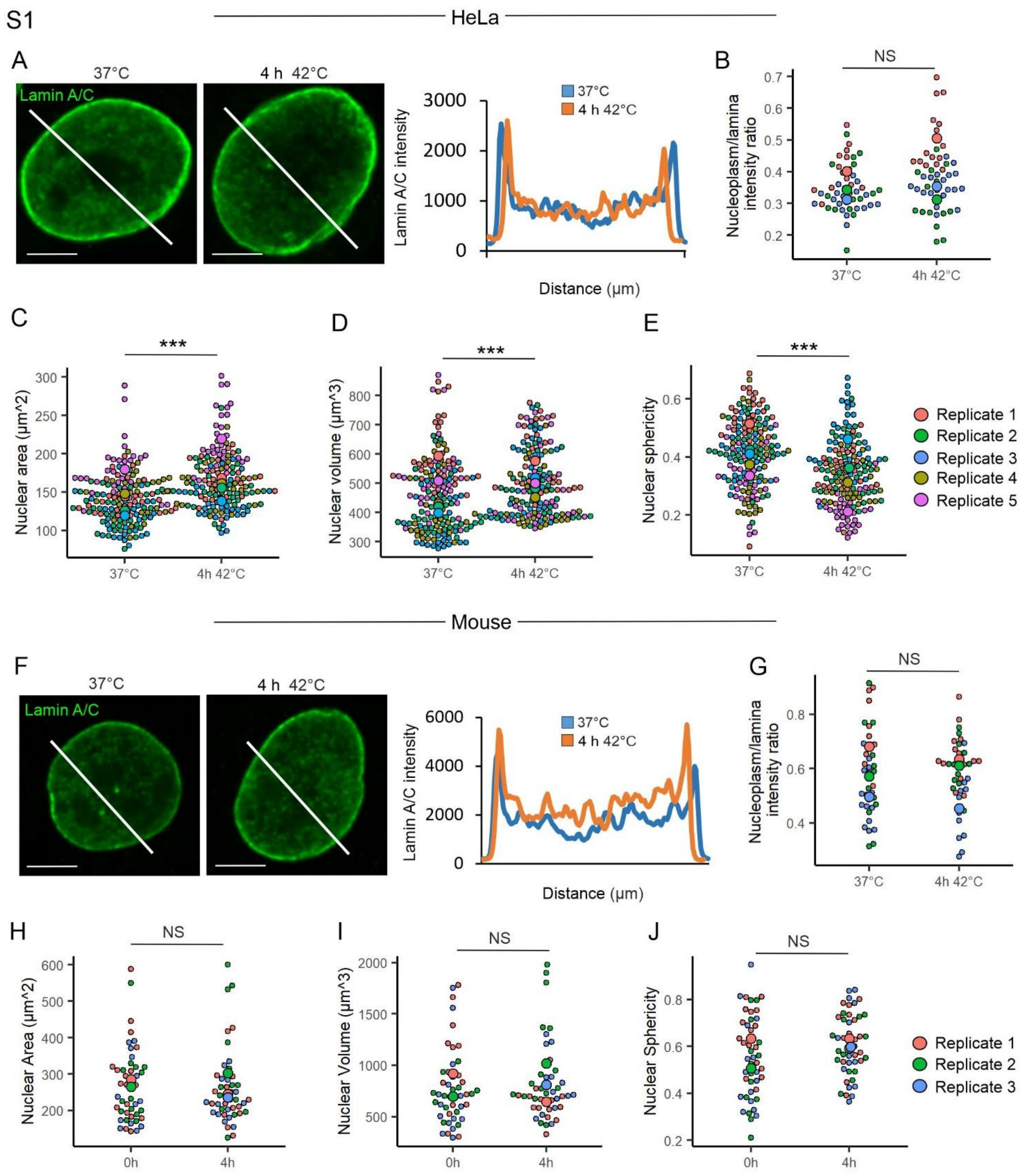
S1
HeLa
A
37°C
4 h 42°C
Lamin A/C
3000
2000
1000
0
Lamin A/C intensity
37°C
4 h 42°C
Distance (µm)
B
NS
Nucleoplasm/lamina intensity ratio
37°C
4h 42°C
C
***
Nuclear area (µm^2)
D
***
Nuclear volume (µm^3)
E
***
Nuclear sphericity
Replicate 1
Replicate 2
Replicate 3
Replicate 4
Replicate 5
Mouse
F
G
H
NS
Nuclear Area (µm^2)
0h
4h
I
NS
Nuclear Volume (µm^3)
J
NS
Nuclear Sphericity

Fig. S1. Morphological changes under mild HS are cell type dependent. A) Representative images of HeLa cells cultured under normal culture conditions or exposed to 4-h HS at 42°C and stained for lamin A/C. The lineplot represent the intensity values of lamin A/C. Scale bar 5 µm. **B)** Intensity values of lamin A/C were measured from lamina and nucleoplasm and the nucleoplasm/lamina intensity ratios were calculated (N=50, from three individual experiment). Data shows individual observations and mean values of each biological replicate, ***p < 0.001 (T-test). **C-E)** Nuclear area (μm^2), volume (μm^3), and sphericity (3D) of HeLa cells (N=150, from five biological replicates). **F)** Representative images of mouse fibroblasts cultured under normal culture conditions or exposed to 4-hour HS at 42°C and stained for lamin A/C. The lineplot represent the intensity values of lamin A/C. **G)** Intensity values of lamin A/C were measured from lamina and nucleoplasm and the nucleoplasm/lamina intensity ratios were calculated (N=40, from three individual experiment). **H-J)** Nuclear area (μm^2), volume (μm^3), and sphericity (3D) of mouse fibroblasts (N=50, from three biological replicates). Data shows individual observations and mean values of each biological replicate, ***p < 0.001 (T-test).

Journal of Cell Science • Supplementary information

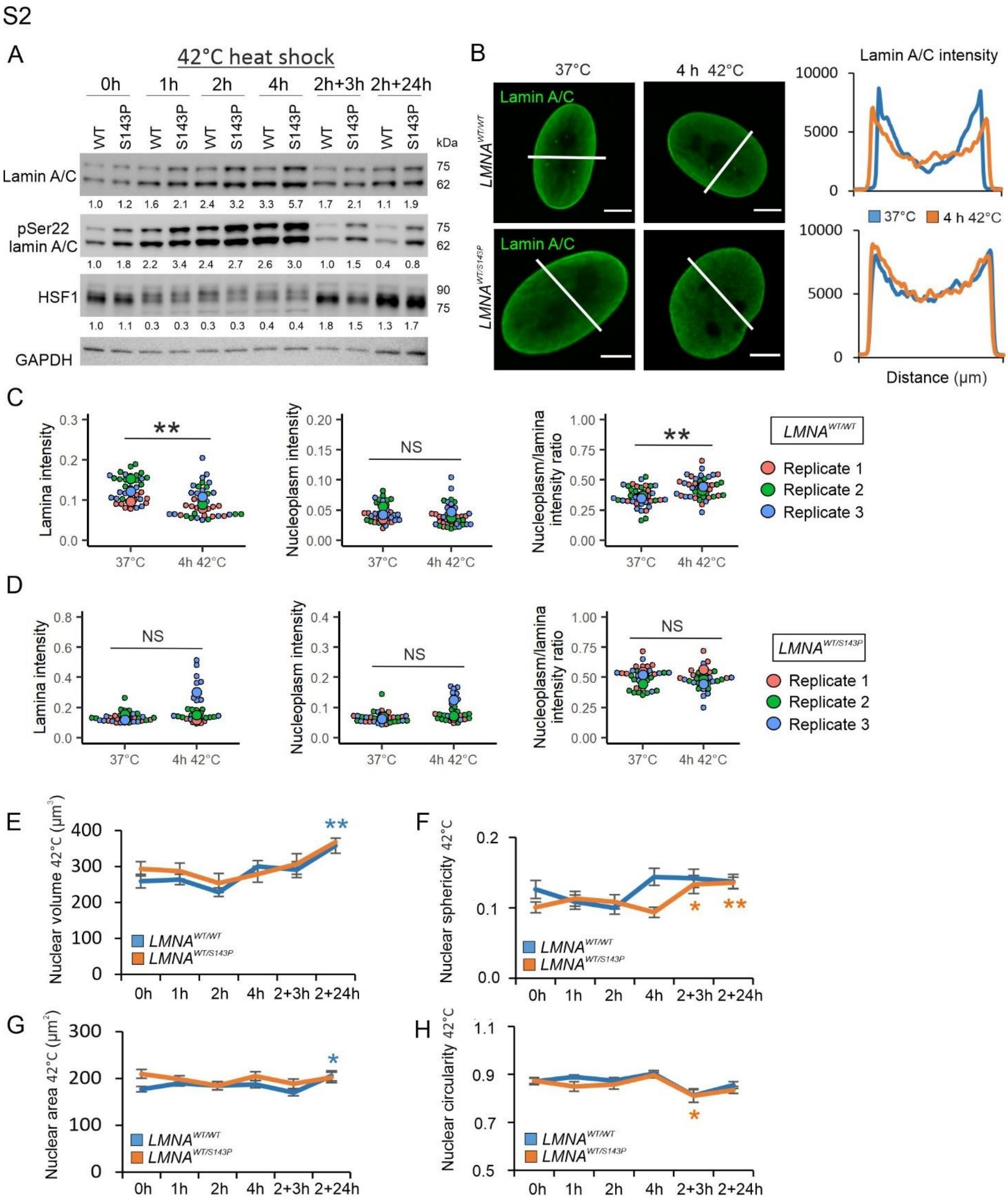

Journal of Cell Science • Supplementary information

Fig. S2. Nuclear morphology is not affected in fibroblasts under mild HS. A) Western blot analysis of control and patient fibroblasts carrying the p.S143P mutation in *LMNA* as detected with lamin A/C, pSer22 lamin A/C and HSF1 antibodies upon 1- to 4-h HS at 42° C and at the recovery. The average numerical values of signal intensities relative to the loading control (GAPDH) are shown below each blot (N=3). pSer22 lamin A/C was normalized to GAPDH and lamin A/C. **B)** Representative images of $LMNA^{WT/WT}$ and $LMNA^{WT/S143P}$ fibroblasts cultured under normal culture conditions or exposed to 4-hour HS at 42°C and stained for lamin A/C. The lineplot represent the intensity values of lamin A/C. **C-D)** Intensity values of lamin A/C were measured from lamina and nucleoplasm and the nucleoplasm/lamina intensity ratios were calculated. The intensity values are normalized to the maximum intensity value ($LMNA^{WT/WT}$: N=40, $LMNA^{WT/S143P}$: N=35, three biological replicates). **E-H)** Nuclear volume (µm^3), sphericity (3D), area (µm^2), and circularity (2D) of $LMNA^{WT/WT}$ and $LMNA^{WT/S143P}$ fibroblasts at different time points of HS at 42°C and at the recovery (N=30, from three biological experiments). Data in C and D shows individual observations and mean values of each biological replicate, lineplots shows mean ± s.e.m., ***$p < 0.001$, **$p < 0.01$, *$p < 0.05$ (T-test).

Journal of Cell Science • Supplementary information

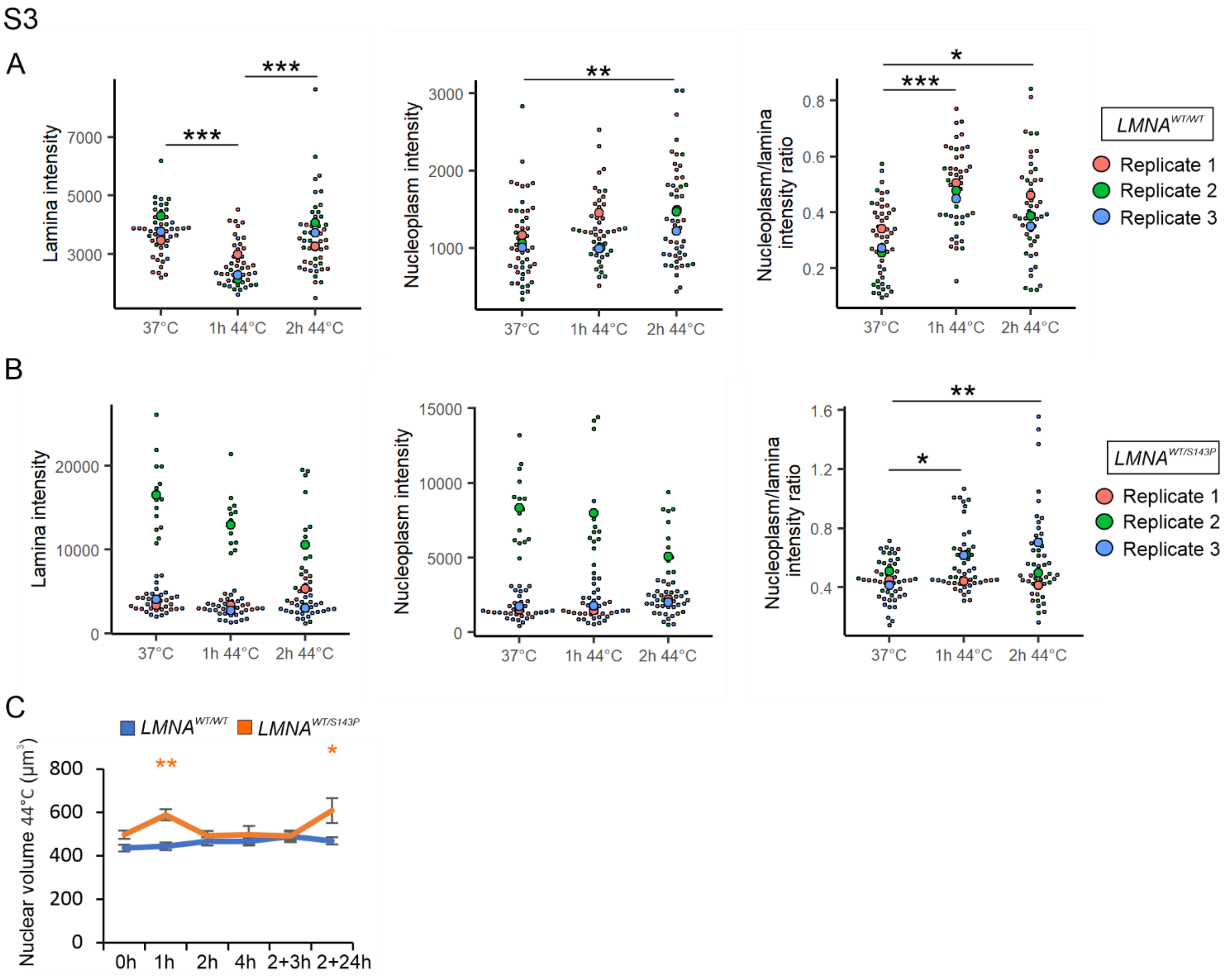

Fig. S3. A fraction of lamin A/C shifts from lamina region into nucleoplasm under severe HS. A-B) Intensity values of lamin A/C were measured from lamina and nucleoplasm and the nucleoplasm/lamina intensity ratios were calculated (N=50, from three individual experiment). Data shows individual observations and mean values of each biological replicate, ***p < 0.001, **p < 0.01, *p < 0.05 (two-way ANOVA/Tukey's post hoc test). **C)** Nuclear volume (µm^3) (N=50, from three biological replicates). Lineplots shows mean ± s.e.m., **p < 0.01, *p < 0.05 (T-test).

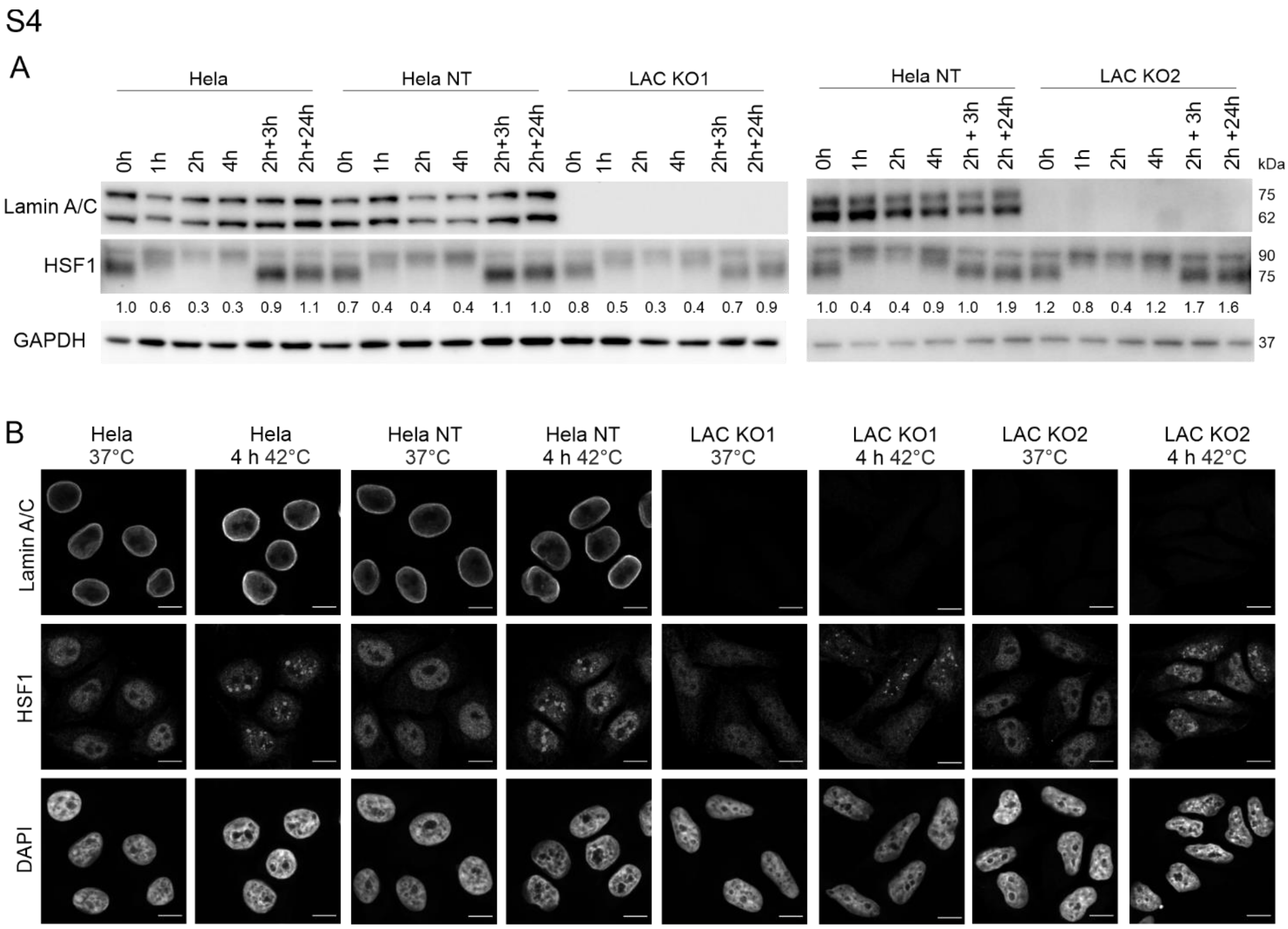

Fig. S4. **Lamin A/C KO does not affect HSF1 expression or localization. A)** Western blot analysis of lamin A/C and HSF1 protein expression at different time points of HS at 42°C and after the recovery. GAPDH was used as a loading control. **B)** Representative images of HeLa, HeLa NT, LAC KO1 and LAC KO2 cells cultured either in normal conditions or exposed to 42°C for 4 h and stained for lamin A/C, HSF1 and DAPI. Scale bar 10 µm.

Journal of Cell Science • Supplementary information

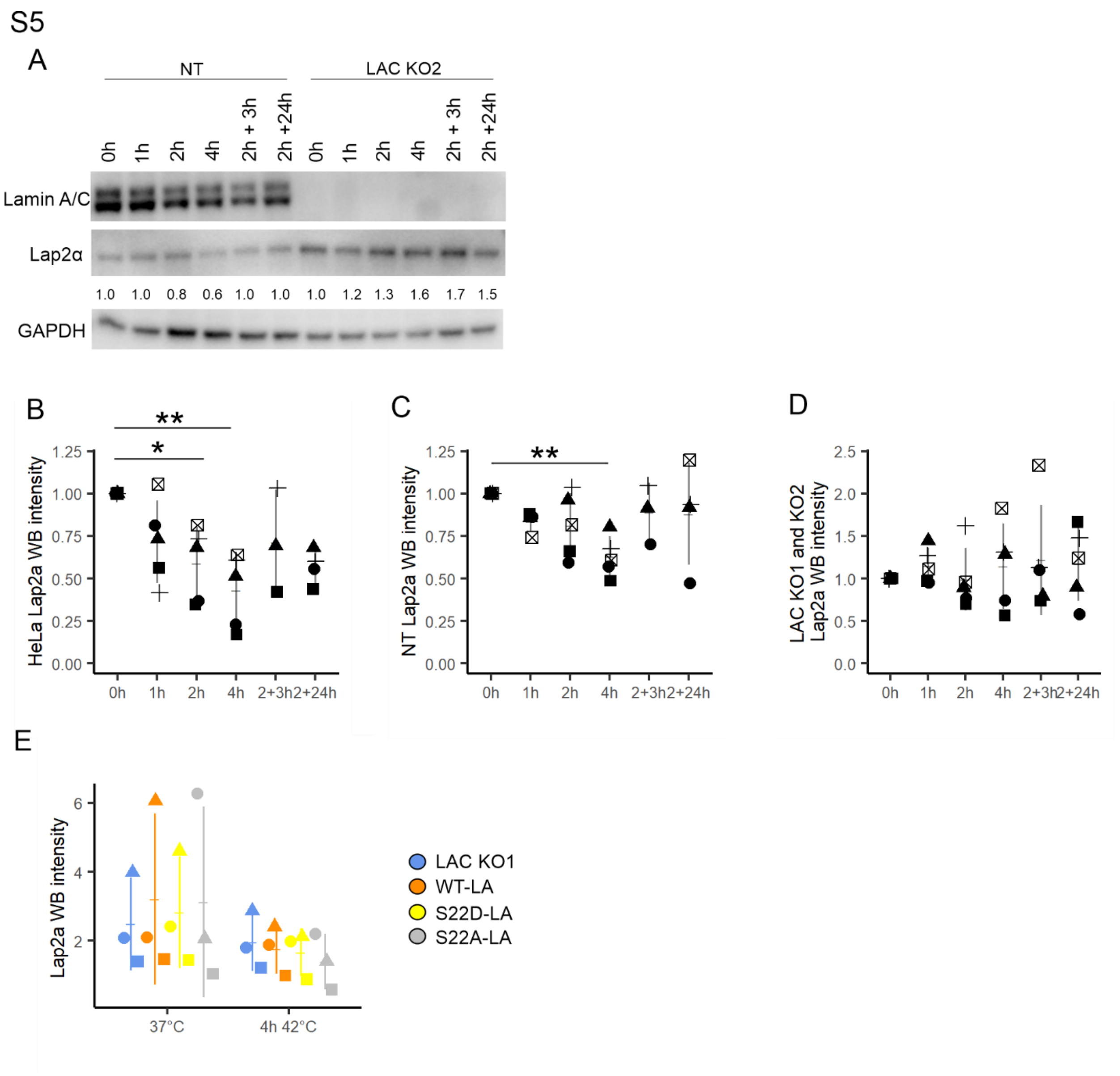

Fig. S5. Lap2α downregulation under HS. A) Western blot analysis of lamin A/C and Lap2α in HeLa NT and LAC KO2 cells at normal culture conditions, after 1- to 4-h HS at 42°C, and at the recovery (N=2). **B)** Lap2α WB intensity values upon HS and recovery in HeLa, NT HeLa and LAC KO1/2 cells (N=5). **C)** Lap2α WB intensity values under normal culture conditions and after 4-hour HS at 42°C (N=3)**.** Whisker plots show mean ± s.d. and individual data points. The shape of the data points indicate each replicate. * $p < 0.05$, ** $p < 0.01$ (Kruskal-Wallis/Dunn's).

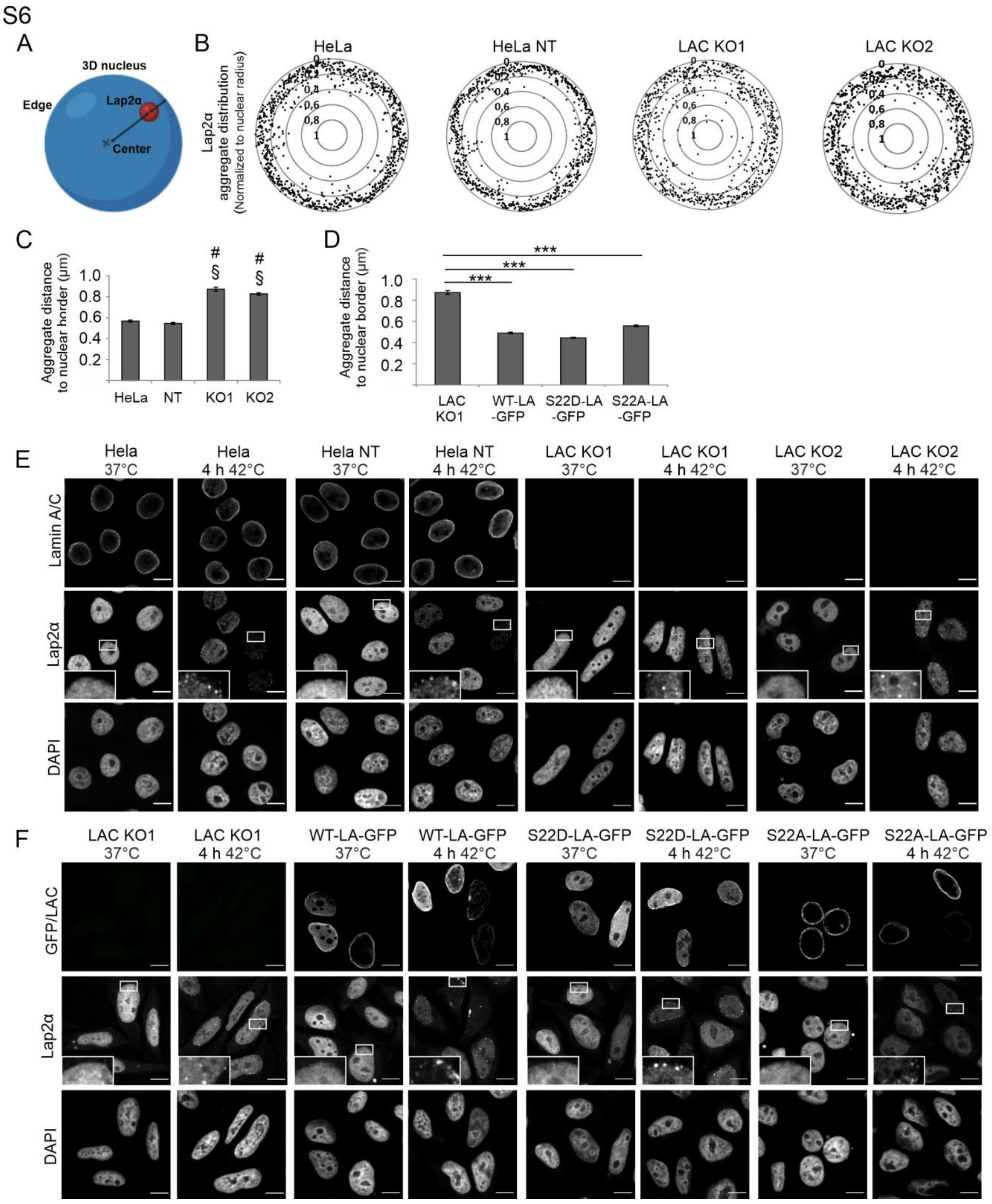
S6
A
3D nucleus
Edge
Lap2α
Center
B
Lap2α aggregate distribution (Normalized to nuclear radius)
HeLa
HeLa NT
LAC KO1
LAC KO2
C
Aggregate distance to nuclear border (µm)
HeLa
NT
KO1
KO2
D
Aggregate distance to nuclear border (µm)
LAC KO1
WT-LA -GFP
S22D-LA -GFP
S22A-LA -GFP
E
Hela 37°C
Hela 4 h 42°C
Hela NT 37°C
Hela NT 4 h 42°C
LAC KO1 37°C
LAC KO1 4 h 42°C
LAC KO2 37°C
LAC KO2 4 h 42°C
Lamin A/C
Lap2α
DAPI
F
LAC KO1 37°C
LAC KO1 4 h 42°C
WT-LA-GFP 37°C
WT-LA-GFP 4 h 42°C
S22D-LA-GFP 37°C
S22D-LA-GFP 4 h 42°C
S22A-LA-GFP 37°C
S22A-LA-GFP 4 h 42°C
GFP/LAC
Lap2α
DAPI

Fig. S6. Distance between Lap2a aggregates and nuclear edge is increased in lamin A/C KO cells. A) ImageJ plugin NucleusJ was used to analyze the distance from Lap2α aggregates (red) to the center and to the edge of the nucleus (as detected by DAPI staining, blue). **B)** Illustration of Lap2α aggregate distribution normalized to nuclear radius (N=1000 aggregates, from three independent experiment). **C-D)** Average distance from the Lap2α aggregates to the edge of the nucleus (N=1000 aggregates, from three individual experiments). **E-F)** Confocal microscopy images showing Lap2α aggregation after 4-h HS at 42°C. The brightness and contrast has been increased in the small insets to visualize the aggregates. Scale bar 10 μm. Data is expressed as mean ± s.e.m, ***p < 0.001, §: compared to HeLa, #: compared to NT HeLa (two-way ANOVA/Tukey' post hoc test).

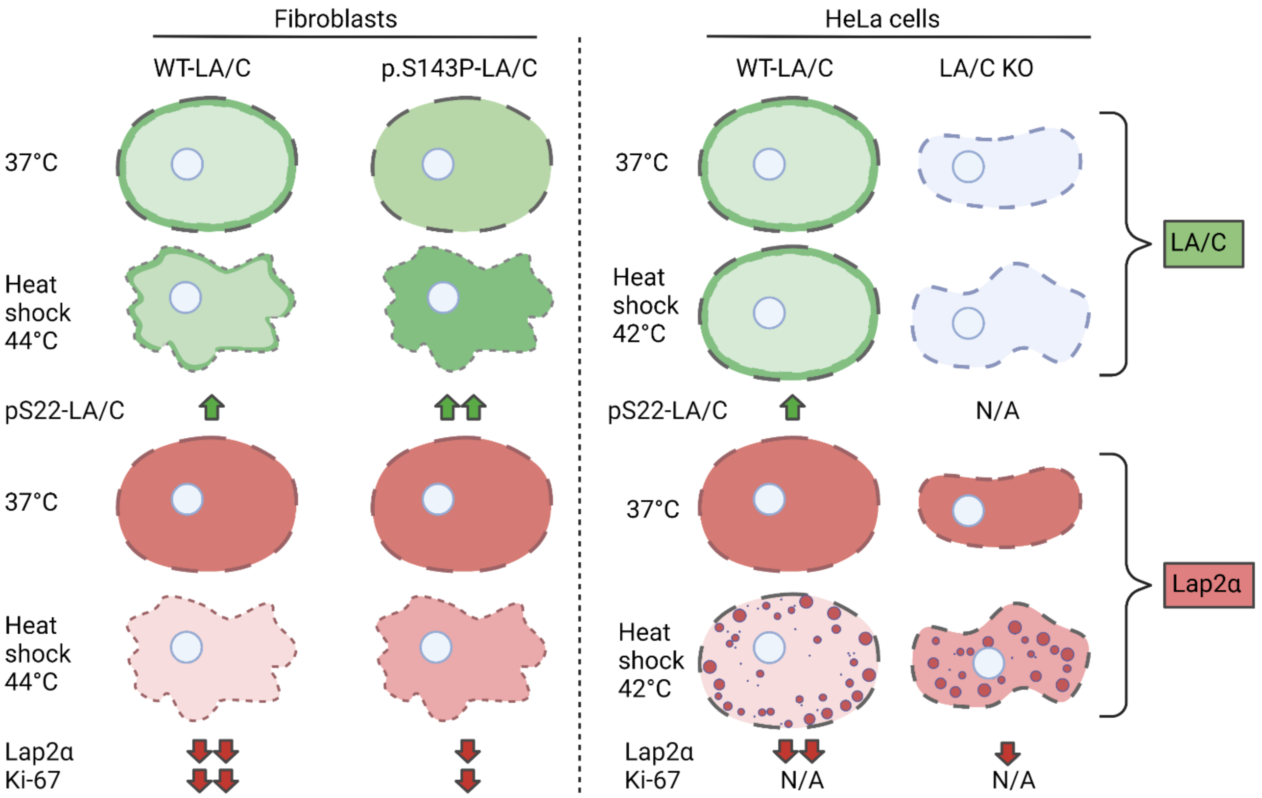

Fig. S7. Schematic illustration of lamin A/C and Lap2α dynamics and nuclear shape changes in human fibroblasts and HeLa cells under HS.

Journal of Cell Science • Supplementary information

Table S1. Lamin A/C phosphorylation sites between control and HS samples identified by LC-MS/MS from interphase Hela cells (N=2).

Position	Phosphopeptide sequence	Phosphorylation sites	Control	2h HS	4h HS
1-7	[-].METPSQR.[R]	T3			x
1-8	[-].METPSQRR.[A]	S5			x
12-25	[R].SGAQASSTPLSPTR.[I]	S12	x	x	x
12-25	[R].SGAQASSTPLSPTR.[I]	T19	x	x	x
12-25	[R].SGAQASSTPLSPTR.[I]	S22	x	x	x
63-72	[R].ITESEEVVSR.[E]	S66	x	x	
90-97	[R].KTLDSVAK.[E]	S94	x	x	x
102-107	[R].LQLELSK.[V]	S107	x		x
266-275	[K].TYSAKLDNAR.[Q]	S268		x	x
271-280	[K].LDNARQSAER.[N]	S277	x	x	x
389-397	[R].LSPSPTSQR.[S]	S390	x	x	x
389-397	[R].LSPSPTSQR.[S]	S392	x	x	x
389-399	[R].LSPSPTSQRSR.[G]	S398		x	
400-417	[R].GRASSHSSQTQGGGSVTK.[K]	S403	x	x	x
402-417	[R].ASSHSSQTQGGGSVTK.[K]	S404	x	x	x
402-417	[R].ASSHSSQTQGGGSVTK.[K]	S407	x	x	x
402-417	[R].ASSHSSQTQGGGSVTK.[K]	S414	x	x	x
421-427	[K].LESTESR.[S]	S423	x	x	x
420-427	[R].KLESTESR.[S]	S426	x	x	x
428-435	[R].SSFSQHAR.[T]	S429	x	x	x
428-435	[R].SSFSQHAR.[T]	S431	x	x	x
456-470	[R].NKSNEDQSMGNWQIK.[R]	S458	x	x	x
585-597	[R].TVLCGTCGQPADK.[A]	T590		x	x
628-634	[R].SVGGSGGGSFGDNLVTR.[S]	S632	x	x	x
628-644	[R].SVGGSGGGSFGDNLVTR.[S]	S636	x	x	x

Journal of Cell Science • Supplementary information
