# Supplementary material for: Lamin A/C phosphorylation at serine 22 is a conserved heat shock response to regulate nuclear adaptation during stress
Source: J Cell Sci. 2023 Feb 27;136(4):jcs259788. doi: 10.1242/jcs.259788 (PMC10022683; doi:10.1242/jcs.259788)
Supplement: Supplementary file 1 [file joces-136-259788_review_history.pdf]

Lamin A/C phosphorylation at serine 22 is a conserved heat shock response to regulate nuclear adaptation during stress

Laura Virtanen, Emilia Holm, Mona Halme, Gun West, Fanny Lindholm, Josef Gullmets, Juho Irjala, Tiina Heliö, Artur Padzik, Annika Meinander, John E. Eriksson and Pekka Taimen
DOI: 10.1242/jcs.259788

Editor: Kathleen Green

Review timeline

Original submission:	19 January 2022
Editorial decision:	28 February 2022
First revision received:	7 August 2022
Editorial decision:	8 September 2022
Second revision received:	12 January 2023
Accepted:	16 January 2023

Original submission

First decision letter

MS ID#: JOCES/2022/259788

MS TITLE: Lamin A/C phosphorylation at serine 22 is a conserved heat shock response to regulate nuclear adaptation during stress

AUTHORS: Laura Virtanen, Emilia Holm, Mona Halme, Gun West, Fanny Lindholm, Josef Gullmets, Juho Irjala, Tiina Helio, Artur Padzik, Annika Meinander, John E Eriksson, and Pekka Taimen
ARTICLE TYPE: Research Article

We have now reached a decision on the above manuscript.

To see the reviewers' reports and a copy of this decision letter, please go to: https://submit-jcs.biologists.org and click on the 'Manuscripts with Decisions' queue in the Author Area. (Corresponding author only has access to reviews.)

As you will see, the reviewers raise a number of substantial criticisms that prevent me from accepting the paper at this stage. They suggest, however, that a revised version might prove acceptable, if you can address their concerns. If you think that you can deal satisfactorily with the criticisms on revision, I would be pleased to see a revised manuscript. We would then return it to the reviewers.

We are aware that you may be experiencing disruption to the normal running of your lab that makes experimental revisions challenging. If it would be helpful, we encourage you to contact us to discuss your revision in greater detail. Please send us a point-by-point response indicating where you are able to address concerns raised (either experimentally or by changes to the text) and where you will not be able to do so within the normal timeframe of a revision. We will then provide further guidance. Please also note that we are happy to extend revision timeframes as necessary.

© 2023. Published by The Company of Biologists under the terms of the Creative Commons Attribution License (https://creativecommons.org/licenses/by/4.0/).

Please ensure that you clearly highlight all changes made in the revised manuscript. Please avoid using 'Tracked changes' in Word files as these are lost in PDF conversion.

I should be grateful if you would also provide a point-by-point response detailing how you have dealt with the points raised by the reviewers in the 'Response to Reviewers' box. Please attend to all of the reviewers' comments. If you do not agree with any of their criticisms or suggestions please explain clearly why this is so.

Reviewer 1

Advance summary and potential significance to field

In the manuscript entitled "Lamin A/C phosphorylation at Ser22 is a conserved heat shock response to regulate nuclear adaptation during stress", work by Virtanen et al highlight a number of exciting facets and significance of lamin Ser22 phosphorylation. The results suggest an evolutionarily conserved mechanism that functions as a protector of the nucleus upon heatshock. These findings reveal the remarkable resilience of not just the nuclear envelope but also the nucleoplasm in responding to stress in a timely manner. It would be interesting to understand the effect of heatshock on the transcriptome of cells and the specific biological pathways that are altered in a lamin dependent manner. The experiments are well designed and performed with relevant controls and the manuscript is certainly suitable for the Journal of Cell Sciences.

Comments for the author

Major comments:

1. The physiological basis for heatshock at 44C is unclear? What is the extent of cell survival/death at 44C after 1h? Is %cell survival significantly different for primary dermal fibroblasts and cells from DCM patients?
2. Fig.1A: The extent of deviation from the average/error from independent western blots is unclear from the mean values below the blots. This data could be included either below the blot or in supplementary figures.
3. Fig.1B: Are the nucleoplasmic & nuclear envelope levels of lamin A/C altered upon heatshock? A quantification would be useful. (ii) Nuclear morphology is altered upon heatshock. While, Hela cells show a distinctively altered nuclear morphology, are similar changes also detected in mouse and human fibroblasts? Is the extent of nuclear morphology changes dependent on the extent to which lamin levels at the nuclear envelope are reduced? Would be useful to include side views (ortho section) of the nucleus to appreciate the extent of altered Z-thickness and nuclear flattening?
4. Fig.2A: From the representative immunoblot, it is rather difficult to ascertain if the basal levels of pSer22 Lamin A/C are significantly elevated? please indicate the extent of deviation/error from the mean calculated from intensities across independent blots.
5. Fig.2C-2D: Although the nucleoplasmic intensity of phosphorylated lamin A/C increased upon heatshock, can this effect be attributed to a redistribution of lamins from the nuclear envelope to the nucleoplasm? In other words, do the level of lamins in the nuclear envelope decrease or remain unaltered? Pl. indicate the number of independent biological replicates from which this data was pooled?
6. While it is interesting that lamin A/C phosphorylation correlates with the rounding up of the nuclei, is such an effect rescued upon Lamin overexpression alone at 44C and to what extent is nuclear morphology restored when cells are brought back to 37C?
7. Fig.3A: Please indicate extent of error quantified from band intensities across independent experiments. It is rather hard to discern how significantly different the levels of pSer22 are?
8. Fig.3D: Please include representative images which show altered heterochromatin staining using DAPI. These differences are not apparent from Fig.3B. Another alternate marker such as HP1a or H3K27me3, could indicate the extent of heterochromatinization.
9. Fig:4; Upon ectopic expression of WT, S22A or S22D to what extent is lamin restored differentially to the nuclear envelope and the nucleoplasm? both in the absence and presence of heatshock?

© 2023. Published by The Company of Biologists under the terms of the Creative Commons Attribution License (https://creativecommons.org/licenses/by/4.0/).

10. Fig.5-7: While the effects of HS are interesting., the significance of the mechanistic connect if any between LaminA/C-HSF1-Lap2a is not quite clear. Please include a schematic/model to explain the potential crosstalk between these factors, which would also complement the discussion nicely.
11. Please mention the number of independent biological replicates from which the nuclei were imaged/quantified, in the figure legends.

Minor Comments:
1. Please check for typos and spelling errors in the manuscript. The manuscript needs careful proofreading and checks for errors.
2. Fig.2 E-I: please change this to more contrasting colours, as the difference between light and dark grey is hard to discern.
3. Please mention in the legend the number of independent biological replicates and technical replicates performed for each experiment.
4. Fig. 2H-I: change " scatter blot" to "scatter plot"

Reviewer 2

Advance summary and potential significance to field

This paper describes essentially a set of experiments that aimed at investigating the effect of heat-shock on nuclei and lamin A/C. I found the paper nice to read. It does incorporate much work on sever experimental set up. Changes of lamin A/C phosphorylation levels and nuclear shape are very interesting.
Therefore, I support the acceptance of the manuscript for publication after considering the points below

Comments for the author

1. It is not clear how the HS conditions were decided. The authors used 4h incubation for 42C, this is much longer than what was used for 44C. Presumably not much alteration occurs at 42C.
2. Line 107. Typo in Ser268
3. The quantifications of the HS nuclei by FM were used statistical tools (e.g. line 137) However, changes in nuclear dimensions requires normalization of the signal. A given nucleoplasmic fluorescence level would increase with nuclear volume decrease (the envelope is proportional to r2 and the volume r3). Namely increase in the intensity of nucleoplasmis lamins over envelope would partially occur due to geometrically consideration and not biologically relevance.
4. It is not clear why two position of CRIPSPR KO were used. Moreover, in some further experiments KO I or two are used. It would be advantages either to use one of the them or both along all experiments.
5. CRIPSPR based KO are better to be characterized to make sure how many nt s were introduced. In any case characterization of these cell lines should be included.
6. What happens to B-type lamins during HS ? Nuclear envelope area is reduced therefore changes in B-type lamins are expected.
7. Differences in Lap2a aggregations between KO1 and KO2 cells are not clear. These cell line should show no statistical significant changes. Isn't suggest that each cell`s procedure would change the results.
8. The observation that Lap2a aggregates and degrade is interesting but is very descriptive and no insights into its meaning are mentioned.
9. Line 37. Changes in lamin structure were not shown in this manuscript

Reviewer 3

Advance summary and potential significance to field

This paper describes for the first time the involvment and consequences of LAC phosphorylation in HS response, which is not only important to understand the mechanism and cellular processes underlying HS, but also to understand the disease causing mechanisms underlying the development of various laminopathies.

© 2023. Published by The Company of Biologists under the terms of the Creative Commons Attribution License (https://creativecommons.org/licenses/by/4.0/).

Comments for the author

In the study by Virtanen et al. the authors investigate the role of A-type lamins in heat shock (HS) response. They show that HS at 42°C for up to 4 hours leads to a gradual increase of lamins A/C phosphorylation at Ser22 in mouse and human cells (and of lamin C at Ser37 in Drosophila) within the nucleoplasm. In addition to Ser22, which is normally phosphorylated during mitosis and involved in lamin disassembly, the authors found five other sites which are differentially phosphorylated upon HS. Upon severe HS (44°C, 1 h) a decrease in nuclear area and an increase in nuclear sphericity was also observed together with an even more dramatic upregulation of Ser22 phosphorylation. Next the authors showed by using specific kinase inhibitors that PKC and MAPK were involved in HS induced Ser22 phosphorylation, but not AKT or CDKs.
Interestingly, downregulation of heat-shock factor 1 (HSF1) leads to reduced lamin A but not lamin C levels and to a general reduction of lamin A/C pSer22 under normal and HS conditions, but the overall kinetics of HS induced Ser22 phosphorylation was not altered compared to WT cells. Beside an increase of LAC pSer22 Virtanen et al. also found a downregulation and redistribution to nuclear aggregates of the A-type lamin specific binding protein LAP2alpha.
Interestingly, the downregulation, which was most likely attributable to protein degragation, was not observable or delayed in LAC KO cells and laminopathic patient cells, respectively. LAP2alpha down-regulation may be attributable to HS-induced cell cycle arrest.

This is a very comprehensive, well carried out and well written study investigating a new conserved mechanism in HS response, phosphorylation of lamin A/C, and its consequences. Therefore I have no major concerns regarding publication in Journal of Cell Science, only minor comments/suggestions which are listed below.

- Regarding the other differentially regulated LAC phosphorylation sites upon HS listed in table S1. Why have they not been detected in control cells in this study but in normal interphase HeLa cells in the Kochin et al. 2014 study?

- Chapter regarding nuclear rounding: In my opinion rounding is a bit misleading as the nuclei become also more misshapen upon 44°C HS. I would stay with sphericity or better circularity, as it was measured in 2D?

- Regarding kinase inhibitors: Does the sentence (line 165-166): "Additionally, MAPK inhibited cells showed significantly less phosphorylation under HS compared to untreated HS cells ($p < 0.05$)." mean, that for Go6976 no increase in lamin phosphorylation upon HS was observed, but this was not statistically significant compared to untreated HS cells?

- The nuclei of shHSF1-cells look smaller than those of the control cells at 37°C and become larger again under HS. They also appear to be less misshapen under HS. Can you comment on this?

- Regarding the LAC knock-out cells: It appears that LB1 is upregulated upon LAC ko. Is this a known phenomenon? In addition, the nuclear shape differs between LAC KO1 (more elongated) and LAC KO2 (more circular and misshapen) cells (Fig. 4D vs. 6D and Fig. S5E). Can you comment on this?

- Figure 4F: LAC KO1 instead of HeLa KO1

- Line 115: Space is missing after the punctuation mark.

- Line 242: I suggest to write "decrease in Lap2α" instead of "Lap2α degradation" as this is shown only later.

- Line 250: Fig. 6F instead of Fig. 6E

- Fig. S5E: some of the magnifications in the inserts (row 2, 3 and 4 don't fit to the actual staining shown in the whole image (intensity and orientation). The inserts are also not mentioned in the figure legend.

© 2023. Published by The Company of Biologists under the terms of the Creative Commons Attribution License (https://creativecommons.org/licenses/by/4.0/).

First revision

Author response to reviewers' comments

Reviewer 1

Major comments:

1. The physiological basis for heat shock at 44C is unclear? What is the extent of cell survival/death at 44C after 1h? Is %cell survival significantly different for primary dermal fibroblasts and cells from DCM patients?

> **Response**: We apologize for not explaining this clearly enough in the original version of the manuscript. While HS at 42°C is in the range of physiological stress that fibroblasts are able to handle, i.e., causing limited signs of exceeded resilience (such as structural changes or cell death), we wanted to use HS at 44°C to determine how the cells respond to more severe stress when the defense mechanisms are exceeded. Indeed, we detected a significant change in the survival, with a ~40% loss of patient cells at the recovery of 48 h as shown in Fig. 7F. In contrast, the WT control cells could handle this severe stress well and showed no significant loss in survival.

2. Fig.1A: The extent of deviation from the average/error from independent western blots is unclear from the mean values below the blots. This data could be included either below the blot or in supplementary figures.

> **Response:** We agree with the reviewer that adding e.g. whisker plots helps with the data interpretation and therefore we have decided to show the individual data points and plots from all the relevant WBs used in the article. The plots from Figure 1A are shown in the 1B.

3. Fig.1B: Are the nucleoplasmic & nuclear envelope levels of lamin A/C altered upon heatshock? A quantification would be useful. (ii) Nuclear morphology is altered upon heatshock. While, Hela cells show a distinctively altered nuclear morphology, are similar changes also detected in mouse and human fibroblasts? Is the extent of nuclear morphology changes dependent on the extent to which lamin levels at the nuclear envelope are reduced? Would be useful to include side views (ortho section) of the nucleus to appreciate the extent of altered Z-thickness and nuclear flattening?

> **Response**: (i) There were no detectable differences in nucleoplasmic and lamina lamin A/C staining intensity ratios between control and heat-shocked HeLa cells (Fig. S1B) or mouse fibroblasts (Fig. S1G). However, $LMNA^{WT/WT}$ human fibroblasts showed a significant decrease in the lamina intensity values after 4-h HS at 42°C (Fig. S2C). We did not detect similar changes in the $LMNA^{WT/S143P}$ fibroblasts (Fig. S2D) and the overall more nucleoplasmic localization of lamin A/C in these cells (due the p.S143P mutation) is likely to explain this difference. (ii) While HeLa cells showed some morphological changes (e.g. area, volume, sphericity) under 4-h HS at 42°C (Fig S1C-E), similar changes were not observed in mouse and human fibroblasts upon 4-h HS at 42°C (Fig. S1H-J, S2E-H).
>
> In summary, there is clearly some variation between different cell lines on how they react on HS at 42°C and fibroblasts appear more resistant compared to epithelial HeLa cells. However, none of the changes are particularly dramatic, which is why we decided to study the morphological changes also during more severe 44°C HS.

© 2023. Published by The Company of Biologists under the terms of the Creative Commons Attribution License (https://creativecommons.org/licenses/by/4.0/).

We have also added the orthogonal views into Figure 1D as suggested by the reviewer.

4. Fig.2A: From the representative immunoblot, it is rather difficult to ascertain if the basal levels of pSer22 Lamin A/C are significantly elevated? please indicate the extent of deviation/error from the mean calculated from intensities across independent blots.

Response: We agree with the reviewer and have now included plots showing deviation and individual data points regarding basal levels of pSer22 Lamin A/C in the control and patient fibroblasts (Fig. 2B). We believe that the difference is now quite clearly illustrated.

5. Fig.2C-2D: Although the nucleoplasmic intensity of phosphorylated lamin A/C increased upon heatshock, can this effect be attributed to a redistribution of lamins from the nuclear envelope to the nucleoplasm? In other words, do the level of lamins in the nuclear envelope decrease or remain unaltered? Pl. indicate the number of independent biological replicates from which this data was pooled?

Response: Indeed, the *LMNA*$^{WT/WT}$ fibroblasts showed significant decrease in the lamina intensity values after 1-h HS at 44°C (Fig. S3A), indicating that there is likely to be a phosphorylation-mediated redistribution of the lamins (which would also be in line with our previous observations; Kochin et al. 2014). After 2 h at 44°C the lamina intensity increased compared to 1 h HS (Fig S3A), but this is likely to be due to increased nuclear wrinkling which intensifies the lamina values. At this time, also the nucleoplasmic lamin A/C increased.

Importantly, the nucleoplasm/lamina intensity ratio values increased significantly in both control and patient fibroblasts after 1-h and 2-h HS which shows a shift in lamin A/C localization upon phosphorylation. These results are from three biological replicates, which are collectively shown in the supplementary Figure S3.

6. While it is interesting that lamin A/C phosphorylation correlates with the rounding up of the nuclei, is such an effect rescued upon Lamin overexpression alone at 44C and to what extent is nuclear morphology restored when cells are brought back to 37C?

Response: Since STA treatment inhibited lamin A/C Ser22 phosphorylation (Fig. 3A) and decreased nuclear rounding (Fig. 3G), it is likely that Ser22 phosphorylation is partially responsible for nuclear rounding process. However, it is possible that there are also other factors involved. Unfortunately, we don't know whether lamin A/C overexpression rescues nuclear rounding at 44°C since transfection efficiency of primary fibroblasts was too low for statistical analysis and HeLa cells are too sensitive for HS at 44°C.

We previously showed nuclear area and sphericity values at the recovery (Fig. 2F-G) and have now added the volume data into Figure S3C. After 24-h recovery, *LMNA*$^{WT/WT}$ fibroblasts restored the normal nuclear area but remained slightly more spherical while the nuclear volume remained constant throughout the experiment (Fig. S3C). In contrast, *LMNA*$^{WT/S143P}$ fibroblasts did not restore the original nuclear area and remained significantly more spherical compared to unheated cells (Fig. 2G). This result shows that mutant cells has decreased capacity to recover from severe stress. Interestingly, the mutant cells also showed increased nuclear volume after 1 h HS and after 24 h recovery (Fig. S3C).

In the revised manuscript, we have added images from 3-h and 24-h recovery phases into the Figure panel 2C.

7. Fig.3A: Please indicate extent of error quantified from band intensities across independent experiments. It is rather hard to discern how significantly different the levels of pSer22 are?

© 2023. Published by The Company of Biologists under the terms of the Creative Commons Attribution License (https://creativecommons.org/licenses/by/4.0/).

Response: We agree with the reviewer and have included plots showing deviation and individual data points (Fig. 3B). The pSer22 lamin A/C intensity change (%) is shown in Figure 3B.

8. Fig.3D: Please include representative images which show altered heterochromatin staining using DAPI. These differences are not apparent from Fig.3B. Another alternate marker such as HP1a or H3K27me3, could indicate the extent of heterochromatinization.

Response: We have carried out repeated experiments with H3K9me3 and H3K27me3 heterochromatin marks to get further insights about chromatin state upon HS. Surprisingly, neither of the markers increased upon HS and it rather seems that H3K9me3 is downregulated (see data below). Since this finding remains unclear and the length of manuscript is around the limit of maximum, we have decided not to speculate HS-induced chromatin changes in the current manuscript and the text has been modified accordingly. More detailed analysis on chromatin state and changes on gene expression will be studied in the follow-up work.

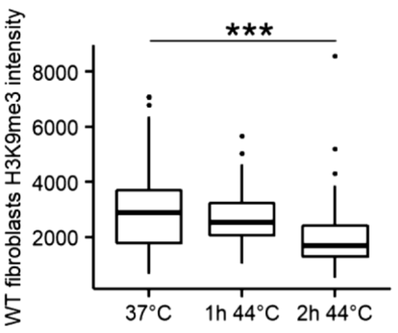

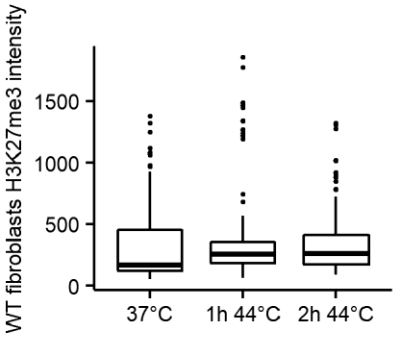

9. Fig.4: Upon ectopic expression of WT, S22A or S22D to what extent is lamin restored differentially to the nuclear envelope and the nucleoplasm? both in the absence and presence of heatshock?

Response: In the absence of HS, the nucleoplasm/lamina-ratio did not differ between WT- LA or S22A-LA expressing cells. However, S22D-LA expressing cells had significantly increased nucleoplasm/lamina-ratio. We want to emphasize that determining the lamina and nucleoplasm intensity values from transfected cells is challenging due to variation in lamin expression levels between the individual cells.

When comparing control and heat-shocked cells, there were no significant changes in nucleoplasm/lamina-ratio between WT-LA or S22A-LA expressing cells, which is compatible with data from untransfected HeLa cells shown in supplementary Figure S1. Surprisingly, the phosphomimetic S22D-LA expressing cells showed slightly decreased nucleoplasm/lamina-ratio under HS (see data below).

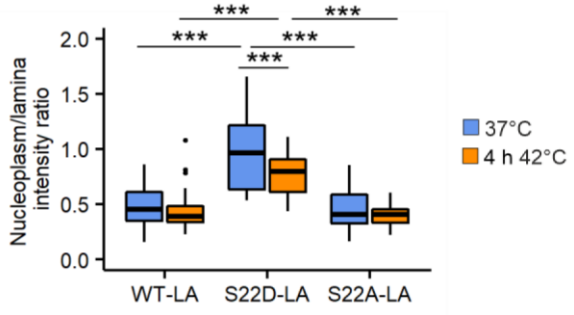

10. Fig.5-7: While the effects of HS are interesting., the significance of the mechanistic connect if any between LaminA/C-HSF1-Lap2a is not quite clear. Please include a schematic/model to explain the potential crosstalk between these factors, which would also complement the discussion nicely.

© 2023. Published by The Company of Biologists under the terms of the Creative Commons Attribution License (https://creativecommons.org/licenses/by/4.0/).

Response: We agree with the reviewer that the crosstalk between lamin A/C-Lap2a-HSF1 and the molecular mechanisms underlying this crosstalk still remain to be elucidated in greater detail, but we feel that it is important to report the included findings. We have done our best to discuss the potential mechanisms in the Discussion part and included a schematic image (Fig. S7, see data below), which summarizes the main findings of the study. We leave it to the editor and the reviewers to decide whether schematic image complements the discussion or should be removed.

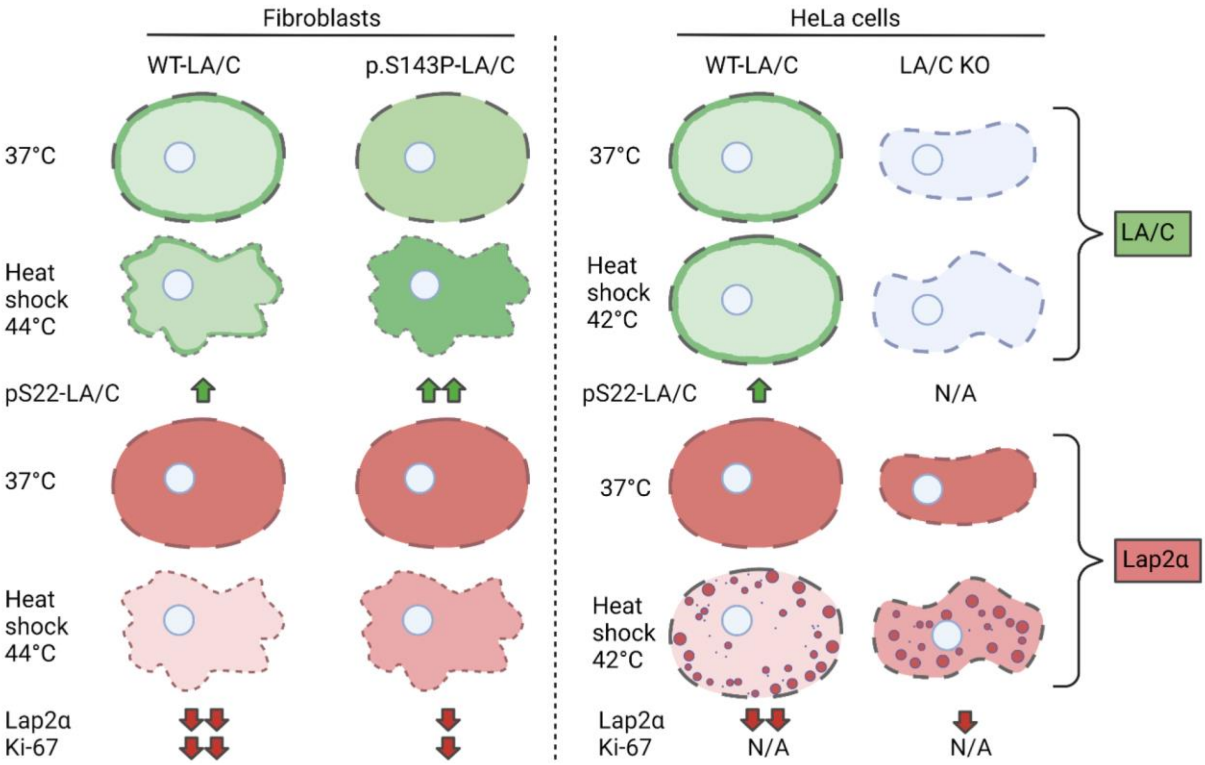

11. Please mention the number of independent biological replicates from which the nuclei were imaged/quantified, in the figure legends.

Response: We apologize for not showing the biological replicates in the original version of the manuscript. We totally agree with the reviewer and have now included the number of biological replicates in all the figure legends.

Minor Comments:

1. Please check for typos and spelling errors in the manuscript. The manuscript needs careful proofreading and checks for errors.

Response: We have carried out a careful proofreading and corrected any typos and spelling errors noticed.

2. Fig.2 E-I: please change this to more contrasting colors, as the difference between light and dark grey is hard to discern.

Response: The colors have been changed.

3. Please mention in the legend the number of independent biological replicates and technical replicates performed for each experiment.

Response: We agree with the reviewer that this is important and have included the number of biological replicates in all the figure legends.

4. Fig. 2H-I: change " scatter blot" to "scatter plot"

© 2023. Published by The Company of Biologists under the terms of the Creative Commons Attribution License (https://creativecommons.org/licenses/by/4.0/).

Response: Corrected.

Reviewer 2 -
1. It is not clear how the HS conditions were decided. The authors used 4h incubation for 42C, this is much longer than what was used for 44C. Presumably not much alteration occurs at 42C.

Response: We apologize for not explaining this clearly enough in the original version of the manuscript. The HS conditions used for HeLa cells (1-4 h at 42°C) are based on the fact that most of the previously published HS studies have been done with shorter time points (0-60min) reflecting the initiation of HS response. We especially wanted to analyse the effects of more prolonged heat shock response on lamin A/C as it has not been studied previously. Additionally, we decided to concentrate more on 4-h timepoint as it showed the most significant increase in pSer22 lamin A/C. However, fibroblasts are more resistant to HS. Since 1-4 h HS at 42°C did not cause detectable morphological changes in fibroblasts, we also studied their response under severe HS (44°C) which clearly enhances the HS response. While HS at 42°C is in the range of physiological stress that fibroblasts are able to handle, i.e. causing limited signs of exceeded resilience (such as structural changes or cell death), 1-2 h HS at 44°C was used to determine how the cells respond to more severe stress when the defence mechanisms are exceeded. We have included a brief description about HS conditions in the revised version of the manuscript (page 5, lines 142-144, 150-151).

2. Line 107. Typo in Ser268

Response: Corrected.

3. The quantifications of the HS nuclei by FM were used statistical tools (e.g. line 137) However, changes in nuclear dimensions requires normalization of the signal. A given nucleoplasmic fluorescence level would increase with nuclear volume decrease (the envelope is proportional to r2 and the volume r3). Namely increase in the intensity of nucleoplasmis lamins over envelope would partially occur due to geometrically consideration and not biologically relevance.

Response: This is an excellent observation. Therefore, we have analyzed nuclear volume, which did not decrease significantly between the timepoints (Fig. S3C). This also reinforces the results that the nucleus becomes more spherical as the area decreases but the volume remains unchanged. We have also changed the pSer22 lamin A/C mean intensity values to Corrected Total Cell Fluorescence (CTCF) values, which takes into account any possible changes in the volume (Fig. 3D-E). Additionally, we have added the intensity values measured from nuclear lamina and nucleoplasm (Fig. S3). These results show that the lamina intensity decreases in control fibroblasts after 1-h HS at 44°C, while nucleoplasmic intensity values remain unaltered. The latter may be due to the analysis method as the nucleoplasmic lamin A/C is only measured from one region of the nucleus instead of the entire nucleus. Alternatively, the nucleoplasmic redistribution may be below our detection limit.

4. It is not clear why two position of CRIPSPR KO were used. Moreover, in some further experiments KO I or two are used. It would be advantages either to use one of the them or both along all experiments.

Response: We apologize for not explaining this clearly enough in the original version of the manuscript. It is strongly advised to use two KO cell clones originating from transductions with different sgRNAs to avoid any results that may appear from off- target effects. All the experiments have been done with both KO cell lines (shown either in the main Figures 4 and 6 or Supplementary Figures S4-S6) with the exception of transfection experiments with different GFP-tagged lamins. We felt comfortable doing the transfections using only one KO clone as all the other experiments showed similar results with both KO clones.

5. CRIPSPR based KO are better to be characterized to make sure how many nt s were introduced. In any case characterization of these cell lines should be included.

© 2023. Published by The Company of Biologists under the terms of the Creative Commons Attribution License (https://creativecommons.org/licenses/by/4.0/).

Response: We are not entirely sure what Reviewer #2 means with this comment. In the case the number of non-targeting (NT) sgRNAs is questioned, we have to admit that only one NT gRNA was used. However, we feel that the original validation of lamin A/C mRNA levels, western blotting and immunofluorescence should be enough to guarantee the feasibility of single cell clones used. Additionally, the sequences of sgRNAs and detailed description of KO cell lines are available in the Materials and methods section.

6. What happens to B-type lamins during HS? Nuclear envelope area is reduced therefore changes in B-type lamins are expected.

Response: We agree with the reviewer that there could be some changes in B-type lamins as there are several published studies that have focused on lamin B1 under HS. However, our study is focused on lamin A/C phosphorylation and therefore the B-type lamins were out of scope of this study, partly because there are no phospho-specific antibodies against B-type lamins available. Furthermore, our previous study on the characterization of phosphorylation-mediated regulation of lamins pointed out that primarily lamin A/C but not lamin B are affected (Kochin et al. 2014).

7. Differences in Lap2a aggregations between KO1 and KO2 cells are not clear. These cell line should show no statistically significant changes. Isn't suggest that each cell`s procedure would change the results.

Response: We are not entirely sure what Reviewer #2 means with this comment. There is virtually no difference in the Lap2a aggregation between the KO1 and KO2 cells and the distance of Lap2a aggregates from the nuclear periphery was highly similar in KO1 and KO2 cells as shown in the Figure S6. The localization of aggregates in KO1 and KO2 cells is, however, statistically significantly different compared to control cells (parental HeLa and NT HeLa) expressing endogenous lamin A/C (as shown in Figure S6C).

8. The observation that Lap2a aggregates and degrade is interesting but is very descriptive and no insights into its meaning are mentioned.

Response: We agree with the Reviewer that this piece of data is descriptive in nature and further studies are needed to find out the detailed mechanism. However, the main focus of the manuscript was on lamin A/C phosphorylation upon HS response and the Lap2a follow-up experiments are out of scope of the current study. In the case the reviewer finds Lap2a data too preliminary and provocative, we are willing to remove the Lap2a data. However, we still think that previously unreported Lap2a aggregation upon HS might be of interest of readers in the current form, especially because it is altered in lamin A/C KO cells.

9. Line 37. Changes in lamin structure were not shown in this manuscript

Response: We apologize for this typo. The word “lamin” has been changed to “lamina”.

Reviewer 3
Minor comments:
1. Regarding the other differentially regulated LAC phosphorylation sites upon HS listed in table S1. Why have they not been detected in control cells in this study but in normal interphase HeLa cells in the Kochin et al. 2014 study?

Response: We thank the reviewer for this relevant comment. We do not know the specific reason for aforementioned difference in repeated experiments but we assume that this may be due to relatively low degree of phosphorylation at corresponding sites and the sensitivity of MS analysis. Previous studies, including the one by Kochin et al. (2014), have shown that most lamin A/C phosphosites are not purely related to certain phase of cell cycle (e.g. mitosis). In other words, the degree of phosphorylation at each site varies depending on conditions and the sites with low phosphorylation status may not become detected in each individual analysis. Nevertheless, our MS analysis suggests that

© 2023. Published by The Company of Biologists under the terms of the Creative Commons Attribution License (https://creativecommons.org/licenses/by/4.0/).

sites other than Ser22 may also become increasingly phosphorylated upon HS when compared to normal conditions.

2. Chapter regarding nuclear rounding: In my opinion rounding is a bit misleading as the nuclei become also more misshapen upon 44°C HS. I would stay with sphericity or better circularity, as it was measured in 2D?

Response: We apologize for not explaining this clearly enough in the original version of the manuscript. The sphericity has been analyzed from 3D images and is shown in Figures 2G. We have also added nuclear volume (Figure 3C) analysis and orthogonal views in the Figure 2 to visualize the changes in the nucleus more clearly.

3. Regarding kinase inhibitors: Does the sentence (line 165-166): "Additionally, MAPK inhibited cells showed significantly less phosphorylation under HS compared to untreated HS cells ($p < 0.05$)." mean, that for Go6976 no increase in lamin phosphorylation upon HS was observed, but this was not statistically significant compared to untreated HS cells?

Response: We agree with reviewer that the exact meaning of the sentence is ambiguous but the indirect conclusion made by the reviewer is correct. Go6976 (PKC inhibitor) treated heat-shocked cells did not show statistically significant difference in Ser22 phosphorylation (increase or decrease) compared to Go6976 treated control cells or to untreated HS cells due to high variation between the biological replications. However, differences in MAPK inhibitor (U0126) and STA treated cells were statistically significant as shown in new Figure 3B.

4. The nuclei of shHSF1-cells look smaller than those of the control cells at 37°C and become larger again under HS. They also appear to be less misshapen under HS. Can you comment on this?

Response: The nuclear area is significantly smaller in shHSF1 cells (Fig. 5C-D) which may be due to smaller overall size of these cells. In addition, the nuclei of both control and shHSF1 cells become larger after HS (Fig. 5C-D and Fig. S1C). We do not know what causes the change in the nuclear area/size in silenced cells or upon HS but this may be related to HSF1-JNK-mTORC1 interplay which has previously been reported to regulate cell and organ size in mice (Su et al., Nat Cell Biol. 2016, 18:527–539). Although these differences are interesting, the main purpose for using shHF1 cells was to test whether HSF1 plays a role in Ser22-lamin A/C phosphorylation, and the specific role of HSF1 in regulating nuclear size is out of scope of the current study.

5. Regarding the LAC knock-out cells: It appears that LB1 is upregulated upon LAC ko. Is this a known phenomenon? In addition, the nuclear shape differs between LAC KO1 (more elongated) and LAC KO2 (more circular and misshapen) cells (Fig. 4D vs. 6D and Fig. S5E). Can you comment on this?

Response: We agree with the reviewer that there appears to be upregulation of lamin B1 in KO1 and KO2 cells when compared to controls. This may be a compensatory mechanism to support the homeostasis at the nuclear envelope although we found no evidence from literature to support this hypothesis.

Two different KO clones were studied to exclude the possibility of any off-target effects. We agree with the reviewer that the nuclear shape slightly differs between the KO lines. Since KO lines have been established from FACS sorted single cells it is possible that differences between individual cells (e.g. composition of cytoskeleton and the amount of structural proteins) eventually affect the morphology of daughter cells. However, despite these slight morphological differences, both KO1 and KO2 cells showed similar results in our studies (e.g. reduced circularity upon HS).

6. Figure 4F: LAC KO1 instead of HeLa KO1

© 2023. Published by The Company of Biologists under the terms of the Creative Commons Attribution License (https://creativecommons.org/licenses/by/4.0/).

Response: Corrected.

7. Line 115: Space is missing after the punctuation mark.

Response: Corrected.

8. Line 242: I suggest to write "decrease in Lap2α" instead of "Lap2α degradation" as this is shown only later.

Response: We agree that this is more logical and we have changed the sentence as suggested.

9. Line 250: Fig. 6F instead of Fig. 6E

Response: Corrected.

10. Fig. S5E: some of the magnifications in the inserts (row 2, 3 and 4 don't fit to the actual staining shown in the whole image (intensity and orientation). The inserts are also not mentioned in the figure legend.

Response: The intensity values have been increased in the inserts to make the aggregates more visible and we have changed the orientation of some of the images to improve the readability. We have also included a description regarding the inserts into Figure legend.

Second decision letter

MS ID#: JOCES/2022/259788

MS TITLE: Lamin A/C phosphorylation at serine 22 is a conserved heat shock response to regulate nuclear adaptation during stress

AUTHORS: Laura Virtanen, Emilia Holm, Mona Halme, Gun West, Fanny Lindholm, Josef Gullmets, Juho Irjala, Tiina Helio, Artur Padzik, Annika Meinander, John E Eriksson, and Pekka Taimen
ARTICLE TYPE: Research Article

We have now reached a decision on the above manuscript.

To see the reviewers' reports and a copy of this decision letter, please go to: https://submit-jcs.biologists.org and click on the 'Manuscripts with Decisions' queue in the Author Area. (Corresponding author only has access to reviews.)

As you will see, the reviewers gave favourable reports but referee #2 re-iterated the importance of validating the cell lines in their comments. I would urge you to include this information in a revised version of the paper. I hope that you will be able to carry this out because I would like to be able to accept your paper, depending on further comments from reviewers.

Please ensure that you clearly highlight all changes made in the revised manuscript. Please avoid using 'Tracked changes' in Word files as these are lost in PDF conversion.

I should be grateful if you would also provide a point-by-point response detailing how you have dealt with the points raised by the reviewers in the 'Response to Reviewers' box. Please attend to all of the reviewers' comments. If you do not agree with any of their criticisms or suggestions please explain clearly why this is so.

© 2023. Published by The Company of Biologists under the terms of the Creative Commons Attribution License (https://creativecommons.org/licenses/by/4.0/).

Reviewer 1

Advance summary and potential significance to field

In this manuscript, the authors for the first time report a unique and novel mechanism of how Lamin A/C phosphorylation at Ser22 regulates adaptation of the nucleus to stress. The study is rigorous and highlights key lamin A/C dependent mechanisms activated upon heatshock.

The detailed edits and corrections to the revised version of the manuscript have greatly enhanced the readability and quality of the manuscript. The authors have satisfactorily addressed the queries from all the 3 reviewers and I recommend the manuscript for publication.

Comments for the author

The manuscript is suitable for publication.

Reviewer 2

Advance summary and potential significance to field

The authors clarified my reservation therefore I suggest to accept the manuscript for publication in J. Cell Science

Comments for the author

Cells should be authenticated. In mots Journal a genetic editing are required to be presented in addition to microscopy and RNA analysis.

Reviewer 3

Advance summary and potential significance to field

This study describes for the first time the involvment and consequences of lamin A/C phosphorylation (at serine 22) as an evolutionary conserved response to heat shock. This is not only important to understand the mechanisms and cellular processes underlying heat shock, but also to understand the disease causing mechanisms leading to the development of various laminopathies.

Comments for the author

This is a well planned, conducted and written study which I think is suitable for publication in JCS.

Second revision

Author response to reviewers' comments

Response to reviewers

Reviewer #2 Comments for the Author:

Cells should be authenticated. In mots Journal a genetic editing are required to be presented in addition to microscopy and RNA analysis.

© 2023. Published by The Company of Biologists under the terms of the Creative Commons Attribution License (https://creativecommons.org/licenses/by/4.0/).

Response:

We have now analyzed the LAC KO1 and KO2 cells with amplicon-based next generation sequencing using MiSeq Illumina platform and identified the genomic alterations of the *LMNA* gene due to CRISPR/Cas9 editing. Below you will find a schematic drawing of the *LMNA* gene showing exons 1-12 (grey boxes), specific sites targeted by sgRNA1 and sgRNA2 (blue lines) and expected amplicons produced by PCR (red lines). MiSeq Illumina sequencing results (lower part of image) show 25 most abundant reads for both LAC KO1 and LAC KO2 cells, edited with sgRNA1 and sgRNA2, respectively. In LAC KO1 cells (on the right), a one nucleotide insertion (adenine) in exon 1 is systematically detected suggesting an identical alteration in both alleles. In LAC KO2 cells (on the left), a one nucleotide insertion (cytosine) and a 20 nucleotide deletion are detected in exon 3 in approximately 1:1 ratio suggesting different alterations in two alleles. No alterations were found in the control HeLa cells treated with non-targeting sgRNA (not shown).

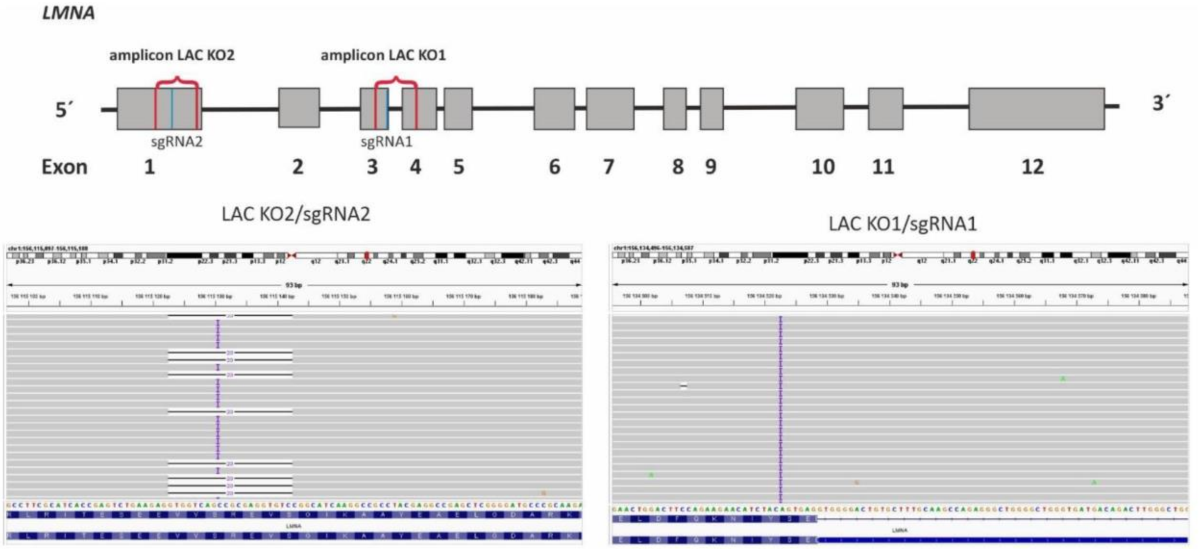

The details regarding CRISPR/Cas9 method and genomic verification of the cells have been added into *Materials and methods* section (lanes 482-510). In addition, we have added a short description of genomic findings into *Results* section (lanes 199-209). Since the number of supplementary figures is already maximum (equal to the total number of main-text figures), the figure above is provided for reviewers'/editor's use only.

<u>Third decision letter</u>

MS ID#: JOCES/2022/259788

MS TITLE: Lamin A/C phosphorylation at serine 22 is a conserved heat shock response to regulate nuclear adaptation during stress

AUTHORS: Laura Virtanen, Emilia Holm, Mona Halme, Gun West, Fanny Lindholm, Josef Gullmets, Juho Irjala, Tiina Helio, Artur Padzik, Annika Meinander, John E Eriksson, and Pekka Taimen
ARTICLE TYPE: Research Article

I am happy to tell you that your manuscript has been accepted for publication in Journal of Cell Science, pending standard ethics checks.

© 2023. Published by The Company of Biologists under the terms of the Creative Commons Attribution License (https://creativecommons.org/licenses/by/4.0/).
